# Investigation of Brønsted acidity in zeolites through adsorbates with diverse proton affinities

**DOI:** 10.1038/s41598-023-39667-5

**Published:** 2023-07-31

**Authors:** Michal Trachta, Ota Bludský, Jan Vaculík, Roman Bulánek, Miroslav Rubeš

**Affiliations:** 1 Institute of Organic Chemistry and Biochemistry, Academy of Sciences of the Czech Republic, Flemingovo nám. 2, 162 10 Prague, Czech Republic; 2 Department of Physical Chemistry, Faculty of Chemical Technology, University of Pardubice, Studentská 573, 532 10 Pardubice, Czech Republic

**Keywords:** Materials science, Materials for energy and catalysis, Porous materials

## Abstract

Understanding the adsorption behavior of base probes in aluminosilicates and its relationship to the intrinsic acidity of Brønsted acid sites (BAS) is essential for the catalytic applications of these materials. In this study, we investigated the adsorption properties of base probe molecules with varying proton affinities (acetonitrile, acetone, formamide, and ammonia) within six different aluminosilicate frameworks (FAU, CHA, IFR, MOR, FER, and TON). An important objective was to propose a robust criterion for evaluating the intrinsic BAS acidity (i.e., state of BAS deprotonation). Based on the bond order conservation principle, the changes in the covalent bond between the aluminum and oxygen carrying the proton provide a good description of the BAS deprotonation state. The ammonia and formamide adsorption cause BAS deprotonation and cannot be used to assess intrinsic BAS acidity. The transition from ion-pair formation, specifically conjugated acid/base interaction, in formamide to strong hydrogen bonding in acetone occurs within a narrow range of base proton affinities (812–822 kJ mol^−1^). The adsorption of acetonitrile results in the formation of hydrogen-bonded complexes, which exhibit a deprotonation state that follows a similar trend to the deprotonation induced by acetone. This allows for a semi-quantitative comparison of the acidity strengths of BAS within and between the different aluminosilicate frameworks.

## Introduction

Aluminosilicate zeolites are an important class of heterogenous catalysts due to the presence of catalytically active acid sites^1,2^. The acid sites are formed upon replacing tetrahedral silicon atom in zeolite lattice with trivalent aluminum and thus introducing a negative charge that needs to be counterbalanced with a cation. Proton compensation results in the formation of Brønsted acid sites (BAS), whereas the presence of metallic cations as compensating species leads to Lewis acid sites (LAS). Also, another source of Lewis acidity is the presence of extra-framework aluminum species (EFAL) that cannot be entirely eliminated during the aluminosilicate synthesis^3^. Understanding the properties and behavior of BAS is essential for optimizing the performance of these heterogeneous catalysts, as the Brønsted acidity is of particular interest due to the wide range of catalytic applications.

**Figure 1:**
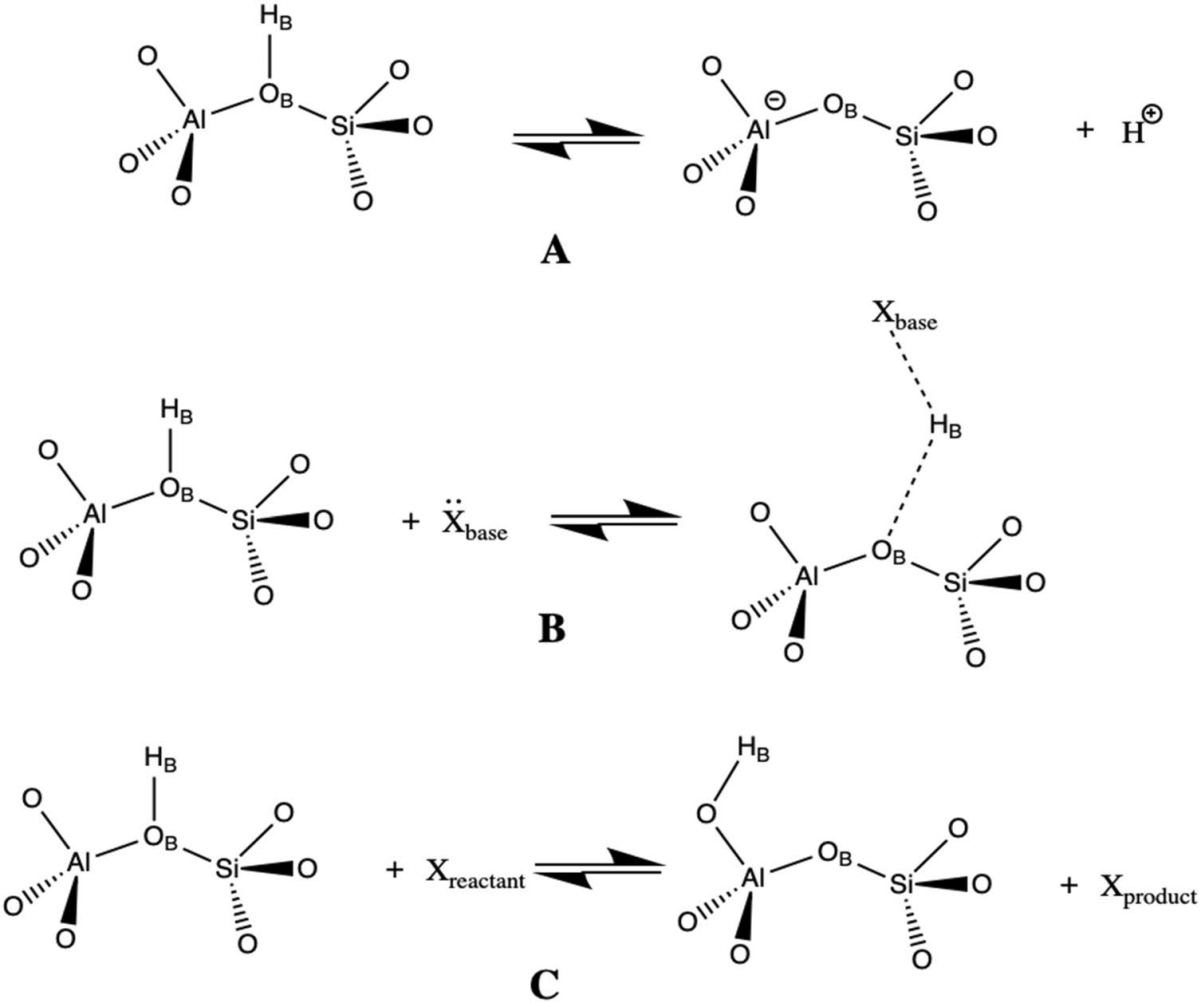
The basic concepts to address acidity of aluminosilicates zeolites (**A**) deprotonation energy, (**B**) adsorption of probe base molecules (e.g. ammonia) and (**C**) test reactions.

During the mid-1990s, significant progress was made in the development of the basic theoretical premises regarding the Brønsted acidity of aluminosilicates^4–7^. These efforts led to several key findings; (i) the IR fingerprint of the bridging hydroxyl band at approximately 3600 cm^−1^ was found to be an inadequate indicator of zeolite acidity strength, in contrast to analogous relationships observed in other acids; (ii) an increase of aluminum content in zeolite framework results in a decrease of zeolite acidity, with the Si/Al ratio limit being topology-dependent; (iii) the proton affinity (PA) of probe base molecules was found to correlate with T–O bond lengths that participate in hydroxyl bond due to the bond order conservation principle; (iv) probe base molecules that deprotonate BAS characterize acid site concentration, rather than the corresponding BAS acidity strength; (v) base probe molecules that form hydrogen bonding with BAS can be used to distinguish acidity strength among the BAS; (vi) acid–base reactions in aluminosilicate materials do not necessarily correlate with the established acidity ranking based on probe adsorption analysis. Figure 1 outlines the fundamental strategies for assessing the BAS strength in aluminosilicate zeolites (or other solid-state acids) by evaluating their ability to donate protons.

The theoretical concept of deprotonation energy (*E*_DPE_) (Fig. 1A) enables to determine the intrinsic acidity of BAS, regardless of the framework’s topology and Si/Al ratio or BAS heterogeneity, even though this quantity is not experimentally observable^8–19^. The main advantage of this approach is that it avoids all BAS accessibility issues related to adsorption. The calculated *E*_DPE_ values indicated that intrinsic BAS acidity weakly correlates with framework density or average isotropic dielectric constant of zeolite. The inclusion of a proton solvation correction in an aluminosilicate medium reveals that the calculated *E*_DPE_ values are relatively consistent (i.e., with a standard deviation of approximately 10 kJ mol^−1^) across various aluminosilicate frameworks. However, calculating the accurate value of *E*_DPE_ is, in principle, quite challenging, especially upon comparing different aluminosilicate zeolites. In addition, *E*_DPE_ appears to be uncorrelated with other acidity descriptors, such as T–O–T angles. Furthermore, efforts to establish a correlation between *E*_DPE_ and ammonia adsorption enthalpies have been unsuccessful, largely due to the formation of hydrogen bonds and dispersion stabilization (i.e., confinement effects) of NH_4_^+^ species within zeolite cavities that render the relation to intrinsic acidity strength hardly tractable. As a result, it was suggested that *E*_DPE_ is an incomplete acidity descriptor without decomposition into covalent and ionic parts and the corresponding analysis of the formed transition state for particular reaction process. In contrast, it was reported that E_DPE_ correlates reasonably well with the adsorption enthalpy of ammonia for the same structural topology but different metal substitutions and similarly for Keggin polyoxometalates.

The adsorption of probe base molecules in zeolites is one of the most used techniques to access aluminosilicate’s acidity and properties (Fig. 1B)^20–30^. Hydrogen-bonded complexes or ionic pairs are formed in dependence on the proton affinity (PA) of the base probe and temperature. Note that the low dielectric constants of zeolites mean that the strength of the probe bases scales more closely with its gas phase values rather than its aqueous counterparts (cf. ammonia and pyridine). The interaction of BAS with weak bases such as carbon monoxide or acetonitrile leads to a significant red shift in BAS ν_OH_ frequency. It has been observed that there is a good correlation between the magnitude of ν_OH_ shift and the corresponding adsorption enthalpy for several aluminosilicate materials^31,32^. This suggests that the ν_OH_ shift could potentially serve as an acidity descriptor. However, this observation was found to be not universally applicable due to the spatial constraints at the BAS, which in turn influence the base orientation and magnitude of the ν_OH_ shift. Similar considerations apply to the frequency shifts on the probe itself (e.g., blue shift in CO frequency)^26^. For strong base probes, the interaction with BAS results in the formation of an ionic pair that can be quantified using experimental techniques such as temperature-programmed desorption (TPD), infrared spectroscopy, and thermogravimetry. However, due to the strong stabilization of the protonated base in the confined space of the aluminosilicate framework, it is difficult to separate the energy requirements of the proton transfer from the overall stabilization energy. As a result, small strong bases like ammonia are useful for sampling the concentration of acid sites, while larger bases like pyridine are better for probing the accessibility of acid sites, but neither samples the intrinsic acidity of the BAS. Additionally, a scaling relation based on the adsorption enthalpies of probe base adsorbates with increasing proton affinity (PA) has been considered as an acidity descriptor. However, while a good correlation between adsorption enthalpies and transition state energies has been observed for certain zeolite topologies, introducing a different topology can clearly break the observed trends.

Besides the standard FT-IR techniques, the solid state magic angle spinning nuclear magnetic resonance (ssMAS NMR) spectroscopy is an extensively used advanced spectroscopy for the study and characterization of acidic sites in solid materials^33–36^. A certain advantage of ssMAS NMR spectroscopy is the possibility to obtain both qualitative and quantitative data on acid centers. For this purpose, a number of nuclei are used, such as ^1^H, ^2^D, ^13^C, ^15^N, ^17^O, ^31^P, etc. The type of hydroxyls on the surface of solids can be distinguished using the chemical shift of ^1^H nuclei ^37^, the accessibility, concentration and reactivity of acid centers can be studied using H/D exchange reactions whose course is monitored using NMR spectroscopy ^38–40^. Adsorption of suitable probe molecules (e.g. pyridine, acetone, acetonitrile, trimethylphosphine (TMP), trimethylphosphine oxide (TMPO), etc.) is often used for determining the acid site strength^41–43^. TMP was found to be insensitive to the strength of the Brønsted acid sites ^44^, while TMPO showed a correlation between the ^31^P chemical shift and the acidic strength of the solid ^45^. Zheng et al. found a linear correlation between δ^31^P chemical shift of TMPO adsorbed and the proton affinity calculated by DFT^43^, and similar correlations were also found for adsorbed pyridine and acetone^46,47^.

The performance of aluminosilicate in a particular reaction or industrial process is of utmost importance (Fig. 1C)^48–60^. A significant observation is that the concentration of Brønsted acidic sites (BAS) is positively correlated with reaction rates. In many processes, there exists a direct relationship between the equilibrium adsorption constant (e.g., Langmuir isotherm model) and acidity strength. Therefore, an aluminosilicate with a lower concentration of highly acidic BAS can exhibit the same activity as a material with a higher concentration of weakly acidic BAS. The optimal test reactions aim to minimize the yield of side products and the effects of diffusion. However, challenges, such as those encountered during the adsorption of probe base adsorptive, persist. One can argue that the complexity increases due to the necessity to form a transition state structure, where the topology of the aluminosilicate can play a significant role (e.g., adsorption on dual cationic sites, promoting the effects of LAS/EFAL).

Assessing the acidity scaling of aluminosilicate BAS remains challenging due to the similarity in the structural motif Si–O_B_ (H_B_)–Al, with structural variations mainly in T–O_B_ bond lengths, T–O_B_–T angle, and proton confinement. Theoretical evaluations are likely to provide a better understanding of BAS properties than experimental measurements, which usually give average values over all available BAS in the material. This study focuses on the structural response of six aluminosilicate frameworks (**FAU**, **CHA**, **IFR**, **MOR**, **FER**, and **TON**) to the increasing proton affinity of probe base adsorbates. The probes range from weaker basicity (acetonitrile and acetone) to stronger base (ammonia), with formamide also investigated to improve understanding of framework response in the narrow range of ionic pair formation (812–854 kJ mol^−1^).

## Methods

The following aluminosilicate zeolites were investigated **FAU**, **CHA**, **IFR**, **MOR**, **FER**, and **TON** (Fig. S1)**.** The BAS were created by replacing each unique Si position with Al, creating negatively charged centers (anions) that were balanced with protons. The structures were optimized with a proton on each symmetrically inequivalent oxygen atoms as defined in a previous study^14^. Note that numbering of T positions and corresponding oxygen labeling follows the IZA notation, except for **TON** material, where lowering of the symmetry leads to two inequivalent O6 oxygens denoted as O6a and O6b^61^. The structures of investigated zeolites were taken from the database of DFT optimized zeolite frameworks^62^. To reduce interactions between neighboring BAS, 1 × 1 × *k* supercells were used for **IFR**, **MOR**, **FER** (*k* = 2), and **TON** (*k* = 3). The modified SLC polarizable force-field in GULP was used for simulated annealing of the structures from 600 K, providing a good starting point for ab initio optimization^63–65^. DFT optimization was performed using the PBE^66^ functional with periodic boundary conditions and PAW pseudopotentials as implemented in the VASP computational package^67–69^. The plane-wave basis energy cutoff was set at 400 eV, and the first Brillouin zone was sampled with the Γ-point, as the investigated zeolites have sufficiently large unit cells. SCF energies and gradients were converged to 10^–7^ eV and 10^–3^ eV/Å, respectively. All the results are summarized in Table S1.

To obtain structures as close to the global minimum as possible, up to 10 different starting positions and orientations were used for optimizing the probe base molecules in the vicinity of the BAS. Note that the initial orientations of probe molecules were constrained so that the atom with lone electron pair (i.e., N/O) was directed at BAS. The adsorption energy, ${E}_{ads}^{BAS}$, is defined as a difference between total energy of the supersystem (zeolite + adsorbate) and both constituting subsystems in their optimized geometries. To obtain adsorption energy at a particular BAS position, the BAS relative stability (${E}_{rel}^{BAS}$≥0 provided in Table S1) needs to be added to the adsorption energy of the most stable BAS:

1 $${-E}_{ads}^{BAS}={E}^{BAS}\left(zeolite+adsorbate\right)- {E}^{BAS}\left(zeolite\right)-E\left(adsorbate\right)+ {E}_{rel}^{BAS}$$

While the importance of dispersion interactions in describing the energetics of adsorption is well known, using dispersion correction to account for structural features of zeolites can be problematic^62^. Especially, upon considering the structural response of the aluminosilicate driven mostly by electrostatic interactions that can be slightly overestimated at the DFT level^25^. Thus, Grimme’s D2 dispersion correction was added a posteriori at the supersystem geometry (i.e., deformation energy is considered only at the PBE level of theory)^70^. Note that the mean dispersion contribution to the adsorption energy is 16% (ammonia), 28% (formamide), 40% (acetone), and 32% (acetonitrile). All regression characteristics are performed iteratively with a reweighted least squares approach to reduce the effect of outliers^71^.

## Results and discussion

The attempts to extract information about the BAS acidity from properties of empty zeolite framework could not establish any relevant link between intrinsic BAS acidity, as determined by deprotonation energy, and proposed descriptors of BAS acid strength (e.g., Al–O_B_–Si angle, see Supplementary Information for more details)^13,19^. Thus, while it would be advantageous to circumvent the intricate behavior of the framework in response to adsorption or the challenges linked to the accessibility of BAS, this study primarily focuses on evaluating acidity via the adsorption of basic probe molecules. The most straightforward descriptor of acidity is the adsorption heat, which provides an exact measure of the BAS response. However, the adsorption heat also includes terms that are completely unrelated to the proton transfer process (see below). If geometric parameters are considered as descriptors, the most natural one would be the O_B_–H_B_ distance (corresponding to the shift in the BAS OH frequency) along with the Al–O_B_–Si angle (i.e., *sp*–*sp*^2^ hybridization). These descriptors may not be optimal in case of (i) “full” proton transfer to the probe molecule and (ii) low framework deformation energy in the vicinity of BAS (e.g., large rings containing BAS). In this work, we propose the descriptor based on monitoring the change in Al-O_B_ bond length upon adsorption, that is normalized by Al–O_B_ change between BAS without an adsorbate (i.e., empty zeolite framework) and the fully deprotonated state (anion in Eq. 2). The advantage of this metric, denoted as *f*_dep_, is that it follows the bond conservation principle, and although the changes invoked by probe base adsorption cannot be, in principle, removed, the response of covalent bond can provide more robust descriptor of acidity than other possible candidates. The degree of deprotonation, as determined by the Al–O_B_ bond lengths, *r*(Al–O_B_), is defined as follows:

2 $${f}_{dep}\left(Al-{O}_{B}\right)=\frac{r(Al-{O}_{B}{)}_{BAS}-r(Al-{O}_{B}{)}_{probe@BAS}}{r(Al-{O}_{B}{)}_{BAS}-r(Al-{O}_{B}{)}_{anion}}$$

### Ammonia adsorption

The adsorption energies and main geometric parameters of NH_3_@BAS complexes are summarized in Table S2. The proton affinity of ammonia (853.6 kJ mol^−1^) is sufficient to remove the proton from the BAS, resulting in the formation of ionic pair with a mean O_B_–H_B_ distance of 1.61 Å. The most stable adsorption complexes do not necessarily form at the lowest energy BAS for any given T position. One reason for this is that the most stable BAS cannot accommodate the NH_3_ molecule (e.g., **TON**/Al1–O2 site, where a BAS-LAS transformation induced by base probes adsorption is observed). Additionally, factors such as diffusion limitations for protonated ammonia can also impact the formation of the most thermodynamically stable NH_3_@BAS complexes. For instance, the most stable BAS at the **MOR**/Al1–O4 position forms a complex in the main channel with a relatively low adsorption energy of 142 kJ mol^−1^. The transition of ammonia to Al1–O2 (BAS with the highest relative energy, cf. Table S1), where NH_4_^+^ is strongly stabilized in the **MOR** side pocket (175 kJ mol^−1^), is not feasible as it would require passing through the **MOR** layer. On the other hand, there are many occurrences when the formation of ionic pairs at the most stable BAS does not preclude the formation of the thermodynamically most stable adsorption minimum at BAS of higher relative energy. Note that the higher energy BAS may become populated upon decreasing the Si/Al ratio^72^. As a result, the observed changes in ammonia adsorption heat upon changes in Si/Al ratio can be to some extent explained by changes in proton distribution and not necessarily significant change in intrinsic BAS acidity (i.e., ammonia still deprotonates the BAS even for low Si/Al ratios). For low Si/Al ratios, a repulsion between ammonia ions can further decrease the adsorption heats. In contrast, it has been shown that the interaction with the nearest neighbor Al atoms can play an important role, and thus a comparison/interpretation of theoretical and experimental values is difficult unless proton distributions along with ammonia loading are treated in the correct statistical fashion for medium to lower Si/Al ratios^15,24,59^.

**Figure 2:**
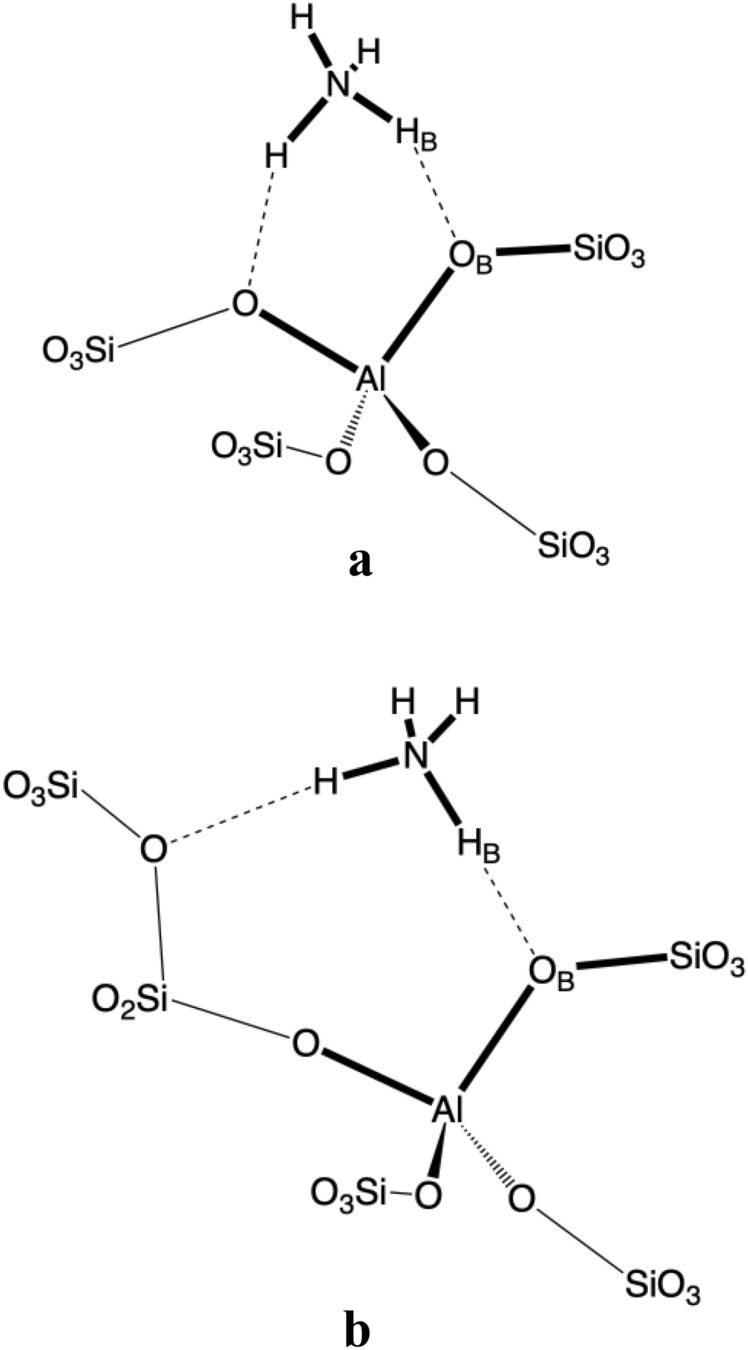
Main structural motifs found in NH_3_@BAS complexes (**a**) two hydrogen bonds with O bonded with Al, and (**b**) one hydrogen bond from original BAS and one with framework oxygen on Si.

Regarding the structural features of NH3@BAS, two main geometry motifs were identified that are consistent with previously determined structures^5,19–21,73,74^. Ammonia forms either two hydrogen bonds with oxygen atoms connected to Al, with one of these bonds usually being shorter (Fig. 2a), or two hydrogen bonds, where one involves the oxygen of original BAS (i.e., Si–O–Al framework oxygen connected to Al) and the second hydrogen bond is with the Si–O–Si framework oxygen (Fig. 2b). These two cases are clearly distinguishable from other reported parameters (e.g., O_B_–H_B_ distance or adsorption energy). The structures like in Fig. 2a yield, on average, significantly smaller O_B_–H_B_–N angles and lower adsorption energies (including dispersion stabilization, see Table S2). Also, the O_B_–H_B_ bond length correlates quite well with the H_B_–N bond length (R^2^ = 0.91). However, the level of correlation with O_B_–N bond length is dependent on the O_B_–H_B_–N angle.

Considering the ammonia adsorption, adsorption energy or enthalpy are typically regarded as primary indicators of BAS acidity. However, the relationship between acidity and adsorption heat is complex due to the dispersion interaction between NH_4_^+^ and the aluminosilicate framework, as well as the formation of hydrogen bonds with framework oxygens^19^. These factors strongly affect the calculated or experimentally measured values, even though they are not directly related to the intrinsic acid strength of the BAS, but rather depend on the framework topology and local framework density. The deprotonation energies do not correlate with any energetic or geometric descriptors of the most stable NH_3_@BAS adsorption complexes in accordance with observation for **MFI**^19^. This is not surprising considering the possibility that small differences in intrinsic acidity between the aluminosilicate’s T-positions are overshadowed by the intermolecular interactions of NH_4_^+^ cation with aluminosilicate. Examining the BAS response to NH_3_ adsorption, the most marked difference is observed in the behavior of the Al–O_B_–Si angle upon comparison with BAS without adsorbate and its corresponding anion. The deprotonation of BAS is accompanied by a substantial decrease in the Al–O_B_–Si angle, but for NH_3_@BAS complexes, the Al–O_B_–Si angle is changed only marginally (−0.2° ± 2.4°). In contrast, upon NH_3_ adsorption, the Al–O_B_ and Si–O_B_ bonds are shortened due to the weakened proton coordination with the framework. This observation also holds for other investigated probes, thus indirectly confirming that the proposed *f*_dep_ descriptor can be used for BAS acidity scaling.

### Formamide adsorption

The results of formamide adsorption with its proton affinity of 822.2 kJ mol^−1^ are summarized in Table S3. The lower proton affinity of formamide is reflected in the fact that, on average, the adsorption energy between formamide and aluminosilicate is lower than that of NH3@BAS by approximately 25 and 10 kJ mol^−1^ at the PBE and PBE-D2 levels of theory, respectively. It appears that the differences in PBE adsorption energies follow more closely the difference in proton affinities. The increase of dispersion interactions, as seen in the case of formamide, decreases the level of correlation between adsorption energy and proton affinity. A similar observation was made in the case of butylamine adsorption, where the significant contribution from dispersion interactions causes the dependence of adsorption enthalpy on proton affinity to deviate from the trend observed with smaller amine probes^21,75^.

**Figure 3:**
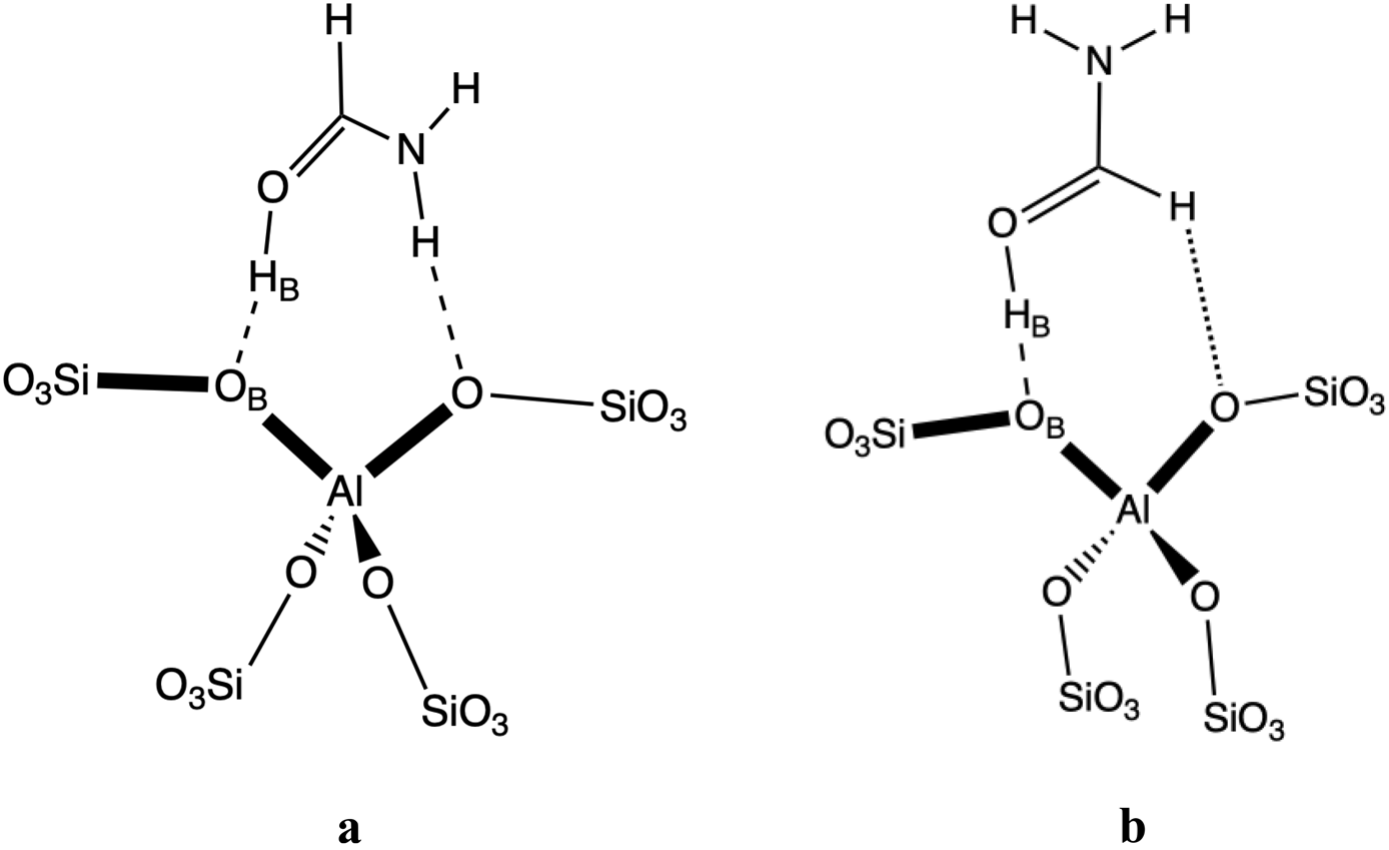
Main structural motifs determined for formamide@BAS adsorption complexes: (**a**) dominant configuration and (**b**) configuration resulting from steric hindrance.

Formamide interacts with BAS preferentially through its carbonyl group rather than its amino group, yielding in most cases BAS deprotonation [i.e., the H_B_–O distance (~ 1.14 Å) is significantly shorter than the O_B_–H_B_ bond length (~ 1.33 Å)]. The most stable formamide@BAS structures usually possess a second strong hydrogen bond, in which the amino group of formamide binds to another framework oxygen connected to Al (Fig. 3a). This structural motif is found in a vast majority of the most stable structures (15 out of the 18 investigated T-positions). BAS that do not permit such a geometrical arrangement due to steric reasons bind formamide only via the carbonyl group (Fig. 3b). In these situations, a weak hydrogen bond is typically formed with the formamide C–H group. Interestingly, if higher energy local minimum structures without additional NH_2_ stabilization are considered, there are about 1/3 structures for which the BAS is not fully deprotonated but rather the proton is shared between the oxygens. This strongly indicates that proton affinity around that of formamide is a borderline region for which the BAS deprotonation occurs. It is straightforward that amino group interaction with another oxygen on the same BAS promotes deprotonation, which is not an optimal behavior required of a “good” base probe^6^. On the other hand, the oxygen from the carbonyl group seems to have a much stringer requirement on the interaction with BAS than nitrogen from the NH_3_ group. This can be immediately seen from the O_B_–H_B_–O (nonlinearity of the hydrogen bond) and H_B_–O–C (enforced by the presence of electron lone pairs on O) angles. These requirements lead to a solid correlation between deprotonation factor *f*_dep_ (Eq. 2) and various bond distances (e.g., O_B_–H_B_, see Fig. S5b).

### Acetone and acetonitrile adsorption

The result of acetone and acetonitrile adsorption are summarized in Tables S4 and S5. There is only a small difference between acetone (PA = 812.0 kJ mol^−1^) and formamide proton affinities of about 10 kJ mol^−1^, but acetone adsorption exhibits rather strong hydrogen bonding than BAS deprotonation or proton-sharing arrangements. The acetonitrile (PA = 779.2 kJ mol^−1^) proton affinity is significantly lower, but it is still sufficient to form strong hydrogen-bonded complexes even on BAS with the intra-zeolitic hydrogen bonds. The comparison between adsorption geometries of carbonyl and nitrile groups shows that while both adsorbates have mean hydrogen bond angles well over 170°, the electron lone pairs on carbonyl oxygen require a narrow range of H_B_–O–C angles, unlike in the case of nitrile group, where larger deviations from the preferable linear arrangement are observed. The acetone/acetonitrile adsorption energies do not correlate with deprotonation energy models. Additionally, in the case of acetone, the *f*_dep_ further correlates with O_B_–H_B_ and other related bond distances similar to the formamide probe. In contrast, no such correlation is observed for the acetonitrile probe (see Fig. S5c, d). The most likely explanation is the lower proton affinity of acetonitrile that introduces significantly smaller variation in O_B_–H_B_ distance than probes of higher PA, and thus the role of local BAS environment plays a more important role. Upon closer examination of Fig. S5, it becomes evident that there is a reasonable correlation between the deprotonation factor (*f*_dep_) and the lengths of the O_B_–H_B_ bonds for base probes that have a PA around the threshold for deprotonation, such as acetone and formamide. However, this correlation disappears for probes with a PA that is too low (acetonitrile) or for probes that induce the deprotonation of the BAS (ammonia). Hence, it can be inferred that the *f*_dep_ descriptor demonstrates the anticipated behavior and appears to be more reliable than relying solely on the O_B_–H_B_ bond lengths, even for base probes that deviate from the optimal proton affinity to a reasonable extent.

### Measuring acidity through adsorbates with diverse proton affinities

**Figure 4:**
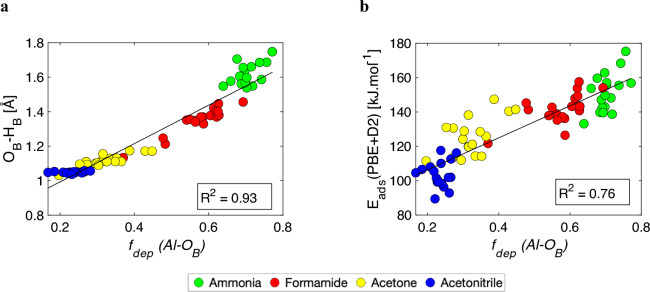
Correlation between deprotonation metric *f*_dep_ as defined in Eq. (2) and O_B_–H_B_ bond lengths (**a**) and adsorption energies (**b**) for the most stable adsorption complexes.

**Figure 5:**
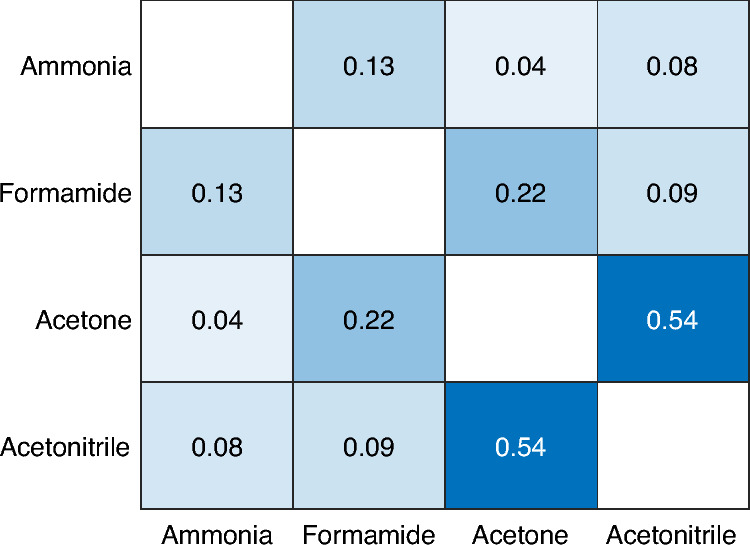
Coefficients of determination (f_dep_ vs f_dep_) indicating a consistency between different probe acidity ranking as determined by the f_dep_ descriptor for the most stable adsorption sites.

Figure 4 shows a dependence of *f*_dep_ (descriptor of acidity) with O_B_–H_B_ distances and adsorption energies for the thermodynamically most stable adsorption sites. The deprotonation factor visibly splits the data into groups according to the proton affinity of the base probe, which is consistent with the response in O_B_–H_B_ bond length and adsorption energy. The inclusion of the dispersion correction diminishes the level of correlation for adsorption energies (cf. Fig. S6), as the dispersion contribution varies with the nature/size of the adsorbate. The trend observed in Fig. 4b illustrates the *E*_ads_–PA relationship, as the correlation between the adsorption energy and *f*_dep_ for a given base probe (with constant PA) is practically nonexistent. Note that Fig. 4 (and Fig. S5b, c) demonstrates a decent correlation between *f*_dep_ and the O_B_–H_B_ bond lengths for base probes with PA close to the deprotonation threshold (O_B_–H_B_ ~ H_B_–X, where X = O, N). However, it does not directly establish a scaling of individual BAS with their acidity strength. To establish such a scaling, the different base probes need to provide reasonably close BAS acidity ranking. Figure 5 presents the coefficients of determination, indicating consistency among different probe acidity rankings as determined by the *f*_dep_ descriptor for the most stable adsorption sites. The data in Fig. 5 clearly demonstrate that each probe yields a slightly different order of BAS acidity ranking. This observation aligns well with the early observation of Lercher et al.^6^, who suggested that the most effective approach to evaluate zeolite acidity is to closely mimic the reactant by using an adsorbate that is as similar as possible. This partially alleviates the effects associated with adsorption that are unrelated to the intrinsic BAS acidity, such as BAS confinement/accessibility and interactions between the probe/adsorbate and the zeolite wall.

**Figure 6:**
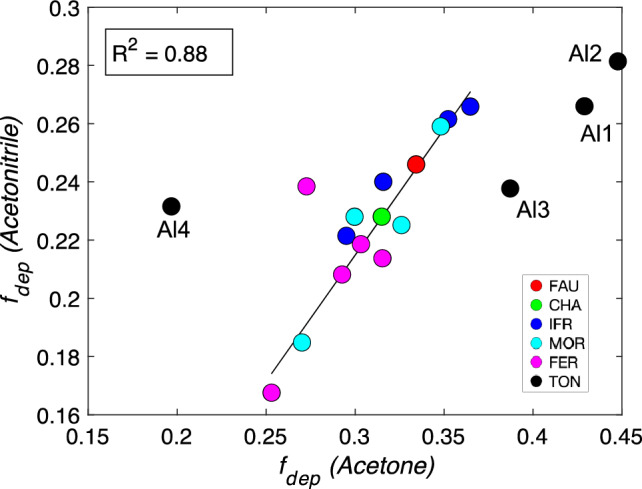
The correlation between deprotonation factors of acetone and acetonitrile.

**Figure 7:**
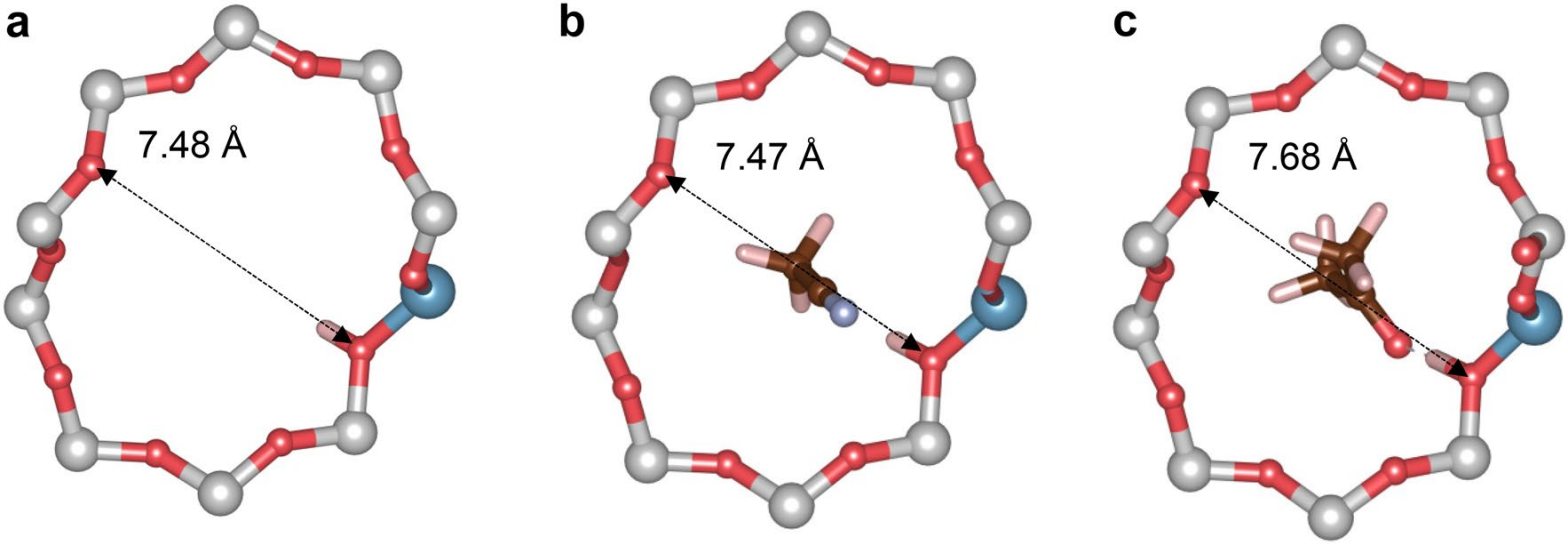
Changes in the 10-ring geometry of **TON**/Al1 (**a**) BAS, (**b**) acetonitrile@BAS, and (**c**) acetone@BAS^76^.

Moreover, Fig. 5 shows that adsorbates that lead to a BAS deprotonation (O_B_–H_B_ > H_B_–X) provide very different rankings from probes with lower proton affinity. This is a result of strong ion-pair interactions, including the formation of hydrogen bonds and dispersion stabilization. It can be concluded that in the case of probes with sufficient proton affinity to “fully deprotonate” the BAS, the information about intrinsic BAS acidity is lost (for more detailed discussion, see Section on *Ammonia Adsorption*). In contrast, there is a weak trend between deprotonation factors of acetone and acetonitrile. The dependence of deprotonation factors for acetone and acetonitrile is depicted in Fig. 6. The obvious outliers (**TON**) demonstrate the difficulties in extracting information about intrinsic acidity from the corresponding deprotonation factors. As mentioned earlier, there is a noticeable difference in the adsorption behavior between acetonitrile and acetone. This disparity arises from the presence of electron lone pairs on the oxygen atom of the carbonyl group, which limits the accessibility of acetone to the BAS. The **TON**/Al4 BAS falls exactly into this category, where the H_B_–O–C angle of about ~ 152° is far from its optimal value of about ~ 120°. Furthermore, the site itself is located deeper within the **TON** layer (Fig. S7), resulting in reduced accessibility of the BAS. This is evident from lower adsorption energies calculated for all the investigated probes. Contradictory to lower adsorption heats, the deprotonation factor of acetonitrile adsorption at **TON**/Al4 BAS does not significantly deviate from the average. The second class of the outliers in Fig. 6 are the remaining **TON** BAS; however, there is no clear indication in the data why this effect is taking place except for significantly longer O_B_–H_B_ bond lengths observed upon acetone adsorption. The comparison of framework geometries reveals that acetone adsorption induces large changes in the 10-ring geometry, indicating that the effect occurs far from the BAS position (see Fig. 7). Interestingly, the shape of the 10-ring changes significantly depending on the position of Al within the ring, suggesting a very high degree of flexibility. For a consistent acidity ranking to be achieved, the base probes must induce a similar framework response. In this case, the acetonitrile has a minimal impact on 10-ring geometry in **TON**/Al1-Al3 except for the local changes at the BAS that are part of that ring. It is straightforward that large differences in framework response to adsorption cause the horizontal shift for **TON**/Al1-3 BAS (only *f*_dep_ for acetone is affected), as observed in Fig. 6.

To summarize, base probes that do not cause deprotonation of the BAS but still possess a sufficiently high proton affinity to bring the proton on the BAS as close as possible to the deprotonation state allow for a semi-quantitative scaling of the BAS acidity between different T-positions within the aluminosilicate framework, as well as between frameworks of different topology. A closer inspection of Fig. 6 indicates that acetonitrile seems to be a superior probe to acetone for the following reasons: (i) acetone requires a stringent geometry arrangement upon adsorption on BAS, (ii) it can cause larger geometry changes in the framework outside the local changes at the BAS most likely due to the presence of two bulky methyl groups. This conclusion seems to be consistent with the observation that acetonitrile bond order parameters obtained from Crystal Orbital Hamilton Population analysis can be used as an indicator of intrinsic acidity strength in **FAU** zeolites^74^.

## Conclusions

Brønsted acidity in zeolites (**FAU**, **CHA**, **IFR**, **MOR**, **FER**, and **TON**) was investigated through the adsorption of base probe molecules (acetonitrile, acetone, formamide, and ammonia) with varying proton affinities. The adsorption complexes displayed interesting characteristics pertaining to the intrinsic acidity of the different Brønsted acid sites (BAS). The transition from the strong hydrogen bonding in acetone to ion-pair formation (i.e., conjugated acid/base interaction) in formamide occurs within a very narrow range of base proton affinities (812–822 kJ mol^−1^). This range appears to be independent of the aluminosilicate material but can be influenced by the accessibility of the BAS or the mode of interaction with the base probe. The degree of BAS deprotonation was assessed through the changes in the Al–O_B_ bond (with the framework oxygen carrying the proton) using the bond order conservation principle.

Base probes leading to full deprotonation of BAS, such as ammonia and formamide, are unsuitable for assessing the intrinsic acidity of these sites, as information about the deprotonation threshold is lost after forming the corresponding ion pair. In contrast, base probes with a proton affinity sufficient to disrupt intra-zeolitic hydrogen bonds but below the deprotonation threshold (Brønsted hydrogen located close to the framework oxygen), such as acetonitrile and acetone, exhibit consistency among their acidity rankings. These rankings are determined by the acidity descriptor (*f*_dep_) for the most stable adsorption sites.

The ranking of BAS is complicated by several factors: (i) small differences in intrinsic acidity among different BAS, as demonstrated by their deprotonation energies, (ii) geometrical constraints arising from the presence of lone pairs on the proton acceptor of the base probe molecule, and (iii) dispersion effects stemming from the framework flexibility, such as deformations of large rings containing BAS. All these issues contribute to the difficulties in extracting information about intrinsic acidity from the corresponding deprotonation factors. Nonetheless, the ranking of BAS obtained from the adsorption of acetonitrile is recommended as the most reliable.

## Supplementary Information

Supplementary Information.

## Data Availability

The datasets used and/or analyzed during the current study available from the corresponding author on reasonable request.

## References

[1] Busca G (2007). Acid catalysts in industrial hydrocarbon chemistry. Chem. Rev..

[2] Derouane EG (2013). The acidity of zeolites: Concepts, measurements and relation to catalysis: A review on experimental and theoretical methods for the study of zeolite acidity. Catal. Rev..

[3] Ravi M, Sushkevich VL, van Bokhoven JA (2020). Towards a better understanding of Lewis acidic aluminium in zeolites. Nat. Mater..

[4] Farneth WE, Gorte RJ (1995). Methods for characterizing zeolite acidity. Chem. Rev..

[5] van Santen RA, Kramer GJ (1995). Reactivity theory of zeolitic Broensted acidic sites. Chem. Rev..

[6] Lercher JA, Gründling C, Eder-Mirth G (1996). Infrared studies of the surface acidity of oxides and zeolites using adsorbed probe molecules. Catal. Today.

[7] Gorte RJ (1999). What do we know about the acidity of solid acids ?. Catal. Lett..

[8] Brand, H. V., Curtiss, L. A. & Iton, L. E. Ab initio molecular orbital cluster studies of the zeolite ZSM-5. 1. Proton affinities. *J. Phys. Chem.* **97**, 12773–12782. 10.1021/j100151a024 (1993).

[9] Kramer GJ, Van Santen RA (1993). Theoretical determination of proton affinity differences in zeolites. J. Am. Chem. Soc..

[10] Eichler U, Brändle M, Sauer J (1997). Predicting absolute and site specific acidities for zeolite catalysts by a combined quantum mechanics/interatomic potential function approach. J. Phys. Chem. B.

[11] Sauer J, Schröder K-P, Termath V (1998). Comparing the acidities of microporous aluminosilicate and silico-aluminophosphate catalysts: A combined quantum mechanics-interatomic potential function study. Collect. Czech. Chem. Commun..

[12] Rybicki M, Sauer J (2015). Acidity of two-dimensional zeolites. Phys. Chem. Chem. Phys..

[13] Rybicki M, Sauer J (2019). Acid strength of zeolitic Brønsted sites—Dependence on dielectric properties. Catal. Today.

[14] Trachta M, Bulanek R, Bludsky O, Rubes M (2022). Bronsted acidity in zeolites measured by deprotonation energy. Sci. Rep..

[15] Grajciar L, Arean CO, Pulido A, Nachtigall P (2010). Periodic DFT investigation of the effect of aluminium content on the properties of the acid zeolite H-FER. Phys. Chem. Chem. Phys..

[16] Deshlahra P, Iglesia E (2016). Toward more complete descriptors of reactivity in catalysis by solid acids. ACS Catal..

[17] Wang CM, Brogaard RY, Weckhuysen BM, Norskov JK, Studt F (2014). Reactivity descriptor in solid acid catalysis: Predicting turnover frequencies for propene methylation in zeotypes. J. Phys. Chem. Lett..

[18] Knaeble W, Carr RT, Iglesia E (2014). Mechanistic interpretation of the effects of acid strength on alkane isomerization turnover rates and selectivity. J. Catal..

[19] Jones AJ, Iglesia E (2015). The strength of Brønsted acid sites in microporous aluminosilicates. ACS Catal..

[20] Boronat M, Corma A (2015). Factors controlling the acidity of zeolites. Catal. Lett..

[21] Boronat M, Corma A (2019). What is measured when measuring acidity in zeolites with probe molecules?. ACS Catal..

[22] Thang HV (2019). The Brønsted acidity of three- and two-dimensional zeolites. Microporous Mesoporous Mater..

[23] Grifoni E (2021). Confinement effects and acid strength in zeolites. Nat. Commun..

[24] Zhao W, Zhang W, Peng S, Liu W, Mei D (2022). Effects of next-nearest-neighbor aluminum location on the Brønsted acidity of HY zeolites. J. Phys. Chem. C.

[25] Rubeš M (2018). Temperature dependence of carbon monoxide adsorption on a high-silica H-FER zeolite. J. Phys. Chem. C.

[26] Arean CO (2014). Measuring the Bronsted acid strength of zeolites—Does it correlate with the O–H frequency shift probed by a weak base?. Phys. Chem. Chem. Phys..

[27] Chakarova K, Hadjiivanov K (2011). Chem. Commun..

[28] Paul G (2018). Combined solid-state NMR, FT-IR and computational studies on layered and porous materials. Chem. Soc. Rev..

[29] Thibault-Starzyk F, Travert A, Saussey J, Lavalley JC (1998). Correlation between activity and acidity on zeolites: A high temperature infrared study of adsorbed acetonitrile. Top. Catal..

[30] Xiao Y (2021). Confinement-driven “flexible” acidity properties of porous zeolite catalysts with varied probe-assisted solid-state NMR spectroscopy. J. Phys. Chem. C.

[31] Khalid M, Makarova MA, Al-Ghefaili KM, Dwyer J (1994). Brønsted acid strength in US-Y: FTIR study of CO adsorption. J. Chem. Soc. Faraday Trans..

[32] Frash MV, Makarova MA, Rigby AM (1997). Quantum-chemical justification of the zeolite acid strength measurement by infrared spectroscopy. J. Phys. Chem. B.

[33] Derouane EG (2013). The acidity of zeolites: Concepts, measurements and relation to catalysis: A review on experimental and theoretical methods for the study of zeolite acidity. Catal. Rev.-Sci. Eng..

[34] Hunger M (1996). Multinuclear solid-state NMR studies of acidic and non-acidic hydroxyl protons in zeolites. Solid State Nucl. Magn. Reson..

[35] Vayssilov GN (2022). Superacidity and spectral signatures of hydroxyl groups in zeolites. Microporous Mesoporous Mater..

[36] Vedrine JC (2015). Acid-base characterization of heterogeneous catalysts: An up-to-date overview. Res. Chem. Intermed..

[37] Medeiros-Costa IC (2021). Silanol defect engineering and healing in zeolites: Opportunities to fine-tune their properties and performances. Chem. Soc. Rev..

[38] Louis B, Walspurger S, Sommer J (2004). Quantitative determination of Bronsted acid sites on zeolites: A new approach towards the chemical composition of zeolites. Catal. Lett..

[39] Blasco T (2010). Insights into reaction mechanisms in heterogeneous catalysis revealed by in situ NMR spectroscopy. Chem. Soc. Rev..

[40] Yang WJ, Wang ZC, Huang J, Jiang YJ (2021). Qualitative and quantitative analysis of acid properties for solid acids by solid-state nuclear magnetic resonance spectroscopy. J. Phys. Chem. C.

[41] Zheng AM, Liu SB, Deng F (2017). P-31 NMR chemical shifts of phosphorus probes as reliable and practical acidity scales for solid and liquid catalysts. Chem. Rev..

[42] Yi XF, Ko HH, Deng F, Liu SB, Zheng AM (2020). Solid-state(31)P NMR mapping of active centers and relevant spatial correlations in solid acid catalysts. Nat. Protoc..

[43] Zheng AM, Liu SB, Deng F (2013). Acidity characterization of heterogeneous catalysts by solid-state NMR spectroscopy using probe molecules. Solid State Nucl. Magn. Reson..

[44] Chu YY (2011). Acidic strengths of Bronsted and Lewis acid sites in solid acids scaled by P-31 NMR chemical shifts of adsorbed trimethylphosphine. J. Phys. Chem. C.

[45] Zheng AM (2008). P-31 chemical shift of adsorbed trialkylphosphine oxides for acidity characterization of solid acids catalysts. J. Phys. Chem. A.

[46] Filek U, Bressel A, Sulikowski B, Hunger M (2008). Structural stability and Bronsted acidity of thermally treated AlPW12O40 in comparison with H3PW12O40. J. Phys. Chem. C.

[47] Fang HJ, Zheng AM, Chu YY, Deng F (2010). C-13 chemical shift of adsorbed acetone for measuring the acid strength of solid acids: A theoretical calculation study. J. Phys. Chem. C.

[48] Lercher, J. A., Jentys, A. & Brait, A. n *Acidity and Basicity Molecular Sieves*. Chap. 17. 153–212 (2008).

[49] Niwa, M. *et al.* Dependence of cracking activity on the Brønsted acidity of Y zeolite: DFT study and experimental confirmation. *Catal. Sci. Technol*. 10.1039/c3cy00195d (2013).

[50] Wang C-M, Brogaard RY, Xie Z-K, Studt F (2015). Transition-state scaling relations in zeolite catalysis: Influence of framework topology and acid-site reactivity. Catal. Sci. Technol..

[51] Wang C, Li S, Mao XY, Caratzoulas S, Gorte RJ (2018). H-D exchange of simple aromatics as a measure of Br Onsted-acid site strengths in solids. Catal. Lett..

[52] Bulanek R, Kubu M, Vaculik J, Cejka J (2019). H/D reactivity and acidity of Bronsted acid sites of MWW zeolites: Comparison with MFI zeolite. Appl. Catal. -Gen..

[53] Čičmanec, P., Kotera, J., Vaculík, J. & Bulánek, R. Influence of substrate concentration on kinetic parameters of ethanol dehydration in MFI and CHA zeolites and relation of these kinetic parameters to acid–base properties. *Catalysts*. 10.3390/catal12010051 (2022).

[54] Verma R, Nair NN (2022). Proton-exchange reaction in acidic zeolites: Mechanism and free energetics. J. Phys. Chem. C.

[55] Zhao R, Haller GL, Lercher JA (2022). Alkene adsorption and cracking on acidic zeolites—A gradual process of understanding. Microporous Mesoporous Mater..

[56] Chizallet C, Bouchy C, Larmier K, Pirngruber G (2023). Molecular views on mechanisms of Bronsted acid-catalyzed reactions in zeolites. Chem. Rev..

[57] Park, H. S. *et al.* Crucial role of alkali metal ions and Si/Al ratio in selective adsorption of 1-octene using faujasite zeolites. *Sep. Purif. Technol.*10.1016/j.seppur.2023.123531 (2023).

[58] Rubeš M (2018). Experimental and theoretical study of propene adsorption on K-FER zeolites: New evidence of bridged complex formation. J. Phys. Chem. C.

[59] Liu C, Li G, Hensen EJM, Pidko EA (2016). Relationship between acidity and catalytic reactivity of faujasite zeolite: A periodic DFT study. J. Catal..

[60] Sastre G (2016). Confinement effects in methanol to olefins catalysed by zeolites: A computational review. Front. Chem. Sci. Eng..

[61] Baerlocher, C. & McCusker, L. B. *Database of Zeolite Structures*. http://www.iza-structure.org/databases/ (http://www.iza-structure.org/databases).

[62] Trachta M, Rubes M, Bludsky O (2022). Toward accurate ab initio modeling of siliceous zeolite structures. J. Chem. Phys..

[63] Sanders, M., Leslie, M. & Catlow, C. Interatomic potentials for SiO_2_. *J. Chem. Soc. Chem. Commun.*10.1039/C39840001271 (1984).

[64] Schröder K-P (1992). Bridging hydrodyl groups in zeolitic catalysts: A computer simulation of their structure, vibrational properties and acidity in protonated faujasites (H–Y zeolites). Chem. Phys. Lett..

[65] Gale, J. D. GULP: A computer program for the symmetry-adapted simulation of solids. *J. Chem. Soc. Faraday Trans.* **93**, 629–637 (1997).

[66] Perdew J, Burke K, Ernzerhof M (1996). Generalized gradient approximation made simple. Phys. Rev. Lett..

[67] Kresse G, Hafner J (1993). Ab initio molecular dynamics for open-shell transition metals. Phys. Rev. B.

[68] Kresse G, Hafner J (1994). Ab-initio molecular-dynamics simulation of the liquid-metal amorphous-semiconductor transition in germanium. Phys. Rev. B.

[69] Kresse G, Joubert D (1999). From ultrasoft pseudopotentials to the projector augmented-wave method. Phys. Rev. B.

[70] Grimme S (2006). Semiempirical GGA-type density functional constructed with a long-range dispersion correction. J. Comput. Chem..

[71] MATLAB v. Version: 9.14.0 (R2023a) (The MathWorks Inc., 2023).

[72] Rubeš, M., Trachta, M., Vaculík, J., Bulánek, R. & Bludský, O. The analysis of the BAS OH band in zeolites. *Microporous Mesoporous Mater.* **13**, 112052 (2022).

[73] Solans-Monfort, X. *et al.* Adsorption of NH(3) and H(2)O in acidic chabazite. Comparison of ONIOM approach with periodic calculations. *J. Phys. Chem. B* **109**, 3539–3545. 10.1021/jp045531e (2005). 10.1021/jp045531e 16851391

[74] Liu C, Tranca I, van Santen RA, Hensen EJM, Pidko EA (2017). Scaling relations for acidity and reactivity of zeolites. J. Phys. Chem. C.

[75] Lee C, Parrillo DJ, Gorte RJ, Farneth WE (1996). Relationship between differential heats of adsorption and Bronsted acid strengths of acidic zeolites: H-ZSM-5 and H-mordenite. J. Am. Chem. Soc..

[76] Momma K, Izumi F (2011). VESTA 3 for three-dimensional visualization of crystal, volumetric and morphology data. J. Appl. Crystallogr..

