# Supplementary material for: Investigation of Brønsted acidity in zeolites through adsorbates with diverse proton affinities
Source: Sci Rep. 2023 Jul 31;13:12380. doi: 10.1038/s41598-023-39667-5 (PMC10390515; doi:10.1038/s41598-023-39667-5)
Supplement: Supplementary file 1 — Supplementary Information. [file 41598_2023_39667_MOESM1_ESM.docx]

Supporting information

Investigation of Brønsted acidity in zeolites through adsorbates with diverse proton affinities

*Michal Trachta^1^, Ota Bludský^1^, Jan Vaculík^2^, Roman Bulánek^2^, Miroslav Rubeš ^1,2*^*

^1^Institute of Organic Chemistry and Biochemistry, Academy of Sciences of the Czech Republic, Flemingovo nám. 2, 162 10 Prague, Czech Republic

^2^Department of Physical Chemistry, Faculty of Chemical Technology, University of Pardubice, Studentská 573, 532 10 Pardubice, Czech Republic

**Figure S1** Investigated zeolite frameworks.^1^

| **FAU** | **CHA** |
| --- | --- |
| 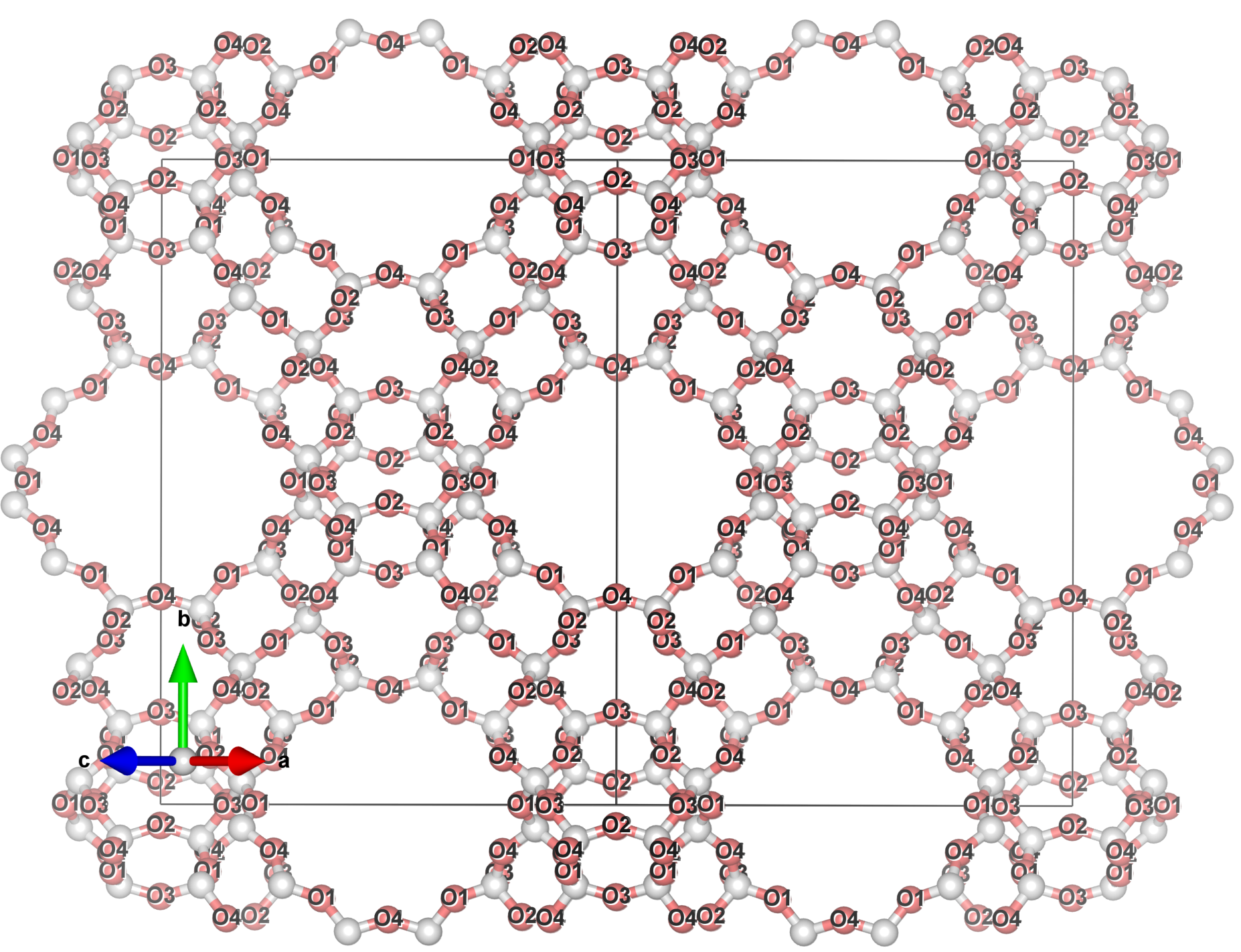 | 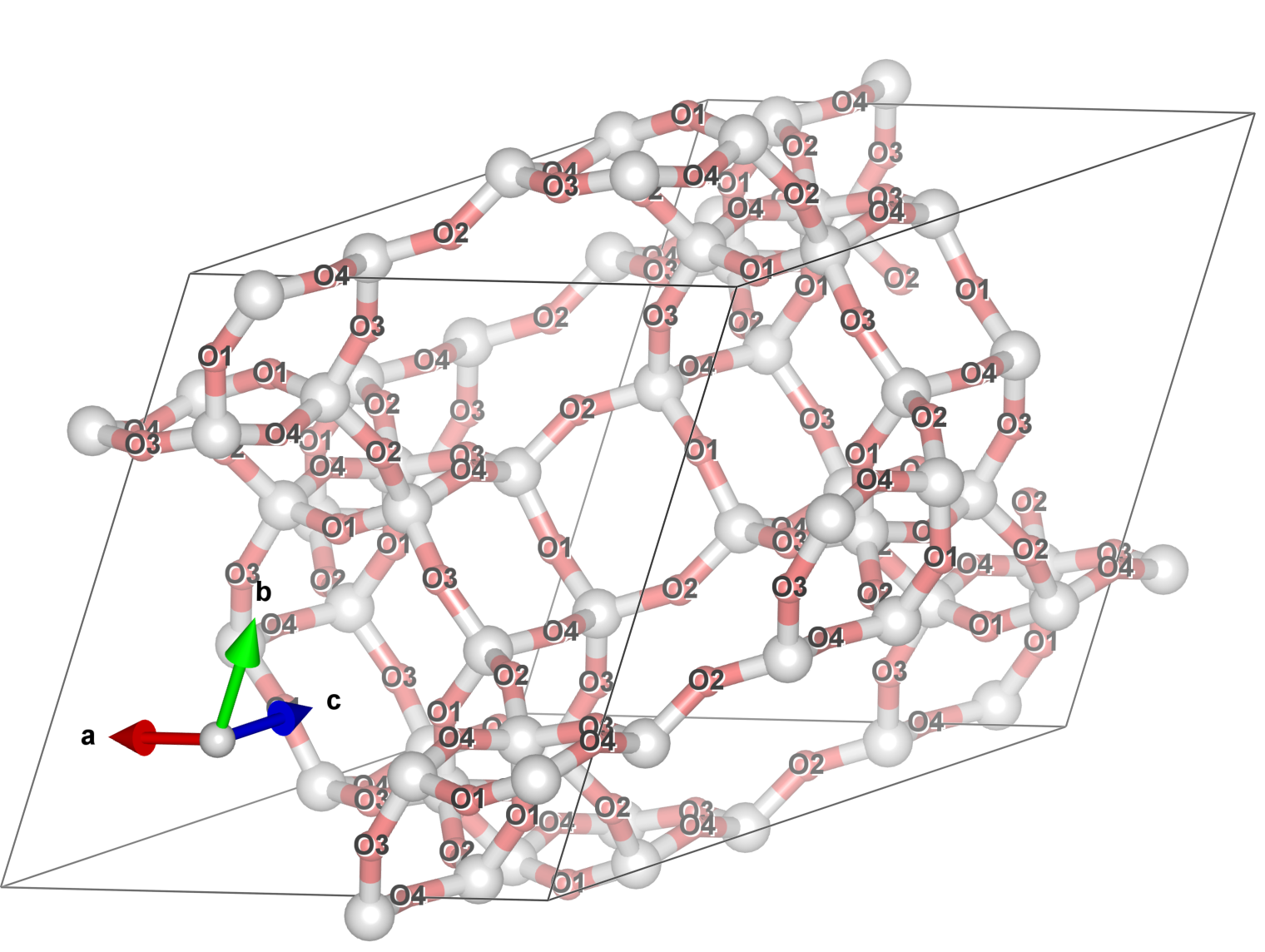 |
| **IFR** | **MOR** |
| 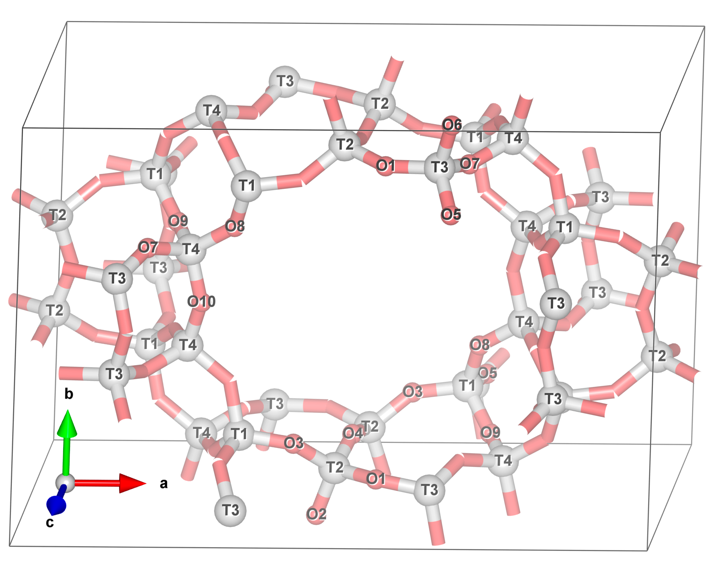 | 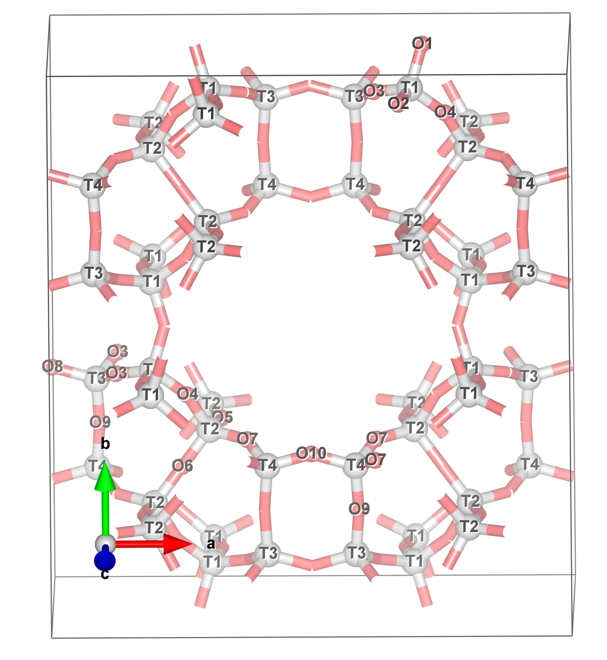 |
| **FER** | **TON** |
| 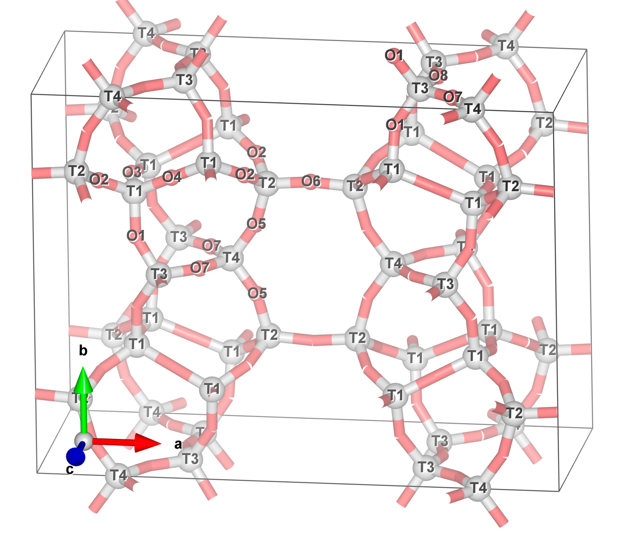 | 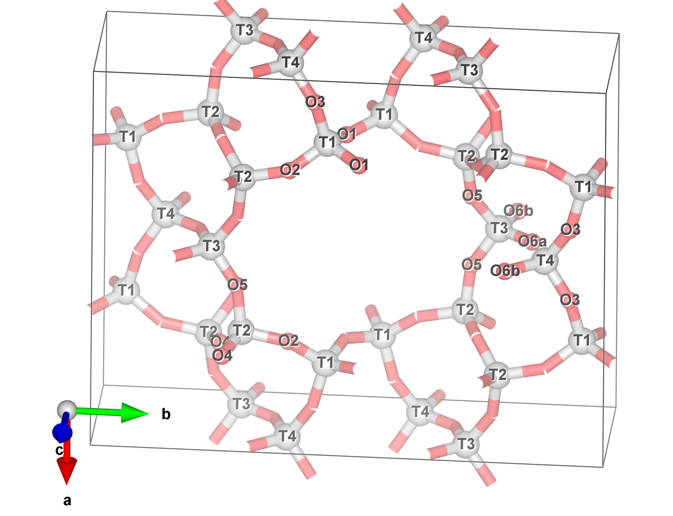 |

**Deprotonation energy**

The deprotonation energy refers to the energy required to remove a proton from the zeolite lattice, as illustrated in Figure 1A of the main article. However, calculating deprotonation energies presents a challenge due to the formation of charged "defects" (i.e., zeolitic anions) and the associated inaccuracies in their energy caused by the introduction of background charge.^2,3^ Resolving this issue can be achieved by employing model cluster calculations, either within the QM/MM approach or by utilizing sufficiently large cluster models, thereby circumventing the limitations imposed by periodic boundary conditions.^4^ Nonetheless, creating cluster models introduces its own set of challenges, as termination effects can influence the deprotonation energy limit.

**Brønsted acid sites without adsorbates**

It was shown that BAS ν_OH_ band and consequently O_B_-H_B_ bond length correlates well with the proton confinement (Figure S2).^2,4^ The proton confinement is defined as follows:^2^

$\eta_{O}=\sum_{i=1}^{N_{O}} \sum_{L} r_{i,L}^{-6}$ (Eq.S1)

The *N*_O_ is the number of oxygen atoms in the unit-cell and second summation runs over the translations of the unit-cell and *r* is the distance between the proton and the framework oxygen. The cut-off radii were set to 30 Å. In addition, the presence of intra-zeolitic hydrogen is assumed when the proton is located within 2.4 Å of a neighboring framework oxygen (O_f_) and the angle O_B_-H_B_-O_f_ is greater than 130 degrees.

**Figure S2** Correlation between proton confinement and r(O_B_-H_B_) bond length for (a) all investigated BAS and (b) BAS, where the intra-zeolitic hydrogen bonded structures were excluded.

The Figure S2a apparently explains why the connection between BAS intrinsic acidity of aluminosilicates and experimentally determined FT-IR ν_OH_ band fingerprint is non-existent. Otherwise, it would imply that BAS with intra-zeolitic hydrogen bond are the most acidic due to the weaker O_B_-H_B_ bond (i.e., lower ν_OH_). This contrasts with energy consideration for the proton transfer to the adsorbate, where this intra-zeolitic hydrogen bond must be broken. Moreover, the BAS with intra-zeolitic hydrogen bonds often undergo blue shift with increasing temperature and the weaker probe bases such as CO not necessarily adsorb on these sites at lower temperatures.^5,6^ It can be seen that the correlation holds even for BAS, where the sites with intra-zeolitic hydrogen bond were excluded (Figure S2b), although the coefficient of determination is slightly lowered. It is possible that for BAS that do not participate in hydrogen bonding, other correlations between local geometry parameters may exist as observed in H-**FER**.^7^ The O_B_-H_B_ bond length, and thus proton confinement (η_O_), correlates reasonably well with Al-O_B_-Si angle (Figure S3). The observed outliers can be attributed to BAS located in 6-membered rings (6MR) that do not conform to the hydrogen-bond criteria (e.g., **IFR**/Al4-O9), and to BAS where aluminosilicate topology results in proton interaction with oxygen on the same Al (e.g., **MFI**/Al4-O19). The observed trend is difficult to interpret in terms of intrinsic BAS acidity because of the two factors. Firstly, if changes in orbital hybridization (i.e., *sp*-*sp*^2^) are primarily responsible for the observed trend, it would follow that Al-O_B_-Si angle is a very good descriptor of intrinsic BAS acidity particularly for higher frequency BAS OH bands. Secondly, there is still a correlation with proton confinement which can cause an adjustment in the Al-O_B_-Si angles due to the flexibility of aluminosilicate lattice (low energy penalty to change T-O-T angles). The latter case is more likely predominant because Al-O_B_-Si angles do not correlate with any deprotonation energy model as proposed in Ref.^3^. A previous study made a similar observation; however, the interpretation was slightly shifted towards observing a very weak trend.^4^

**Figure S3** Correlation between r(O_B_-H_B_) and Al-O_B_-Si angle for BAS with excluded intra-zeolitic hydrogen bonds.

The bond order conservation principle reflects the changes going from the deprotonated state (i.e., conjugated base $\mathrm{AlO}_{4}^{-}$ ) to BAS. Thus, T-O_B_ bond lengths and Al-O_B_-Si angle quite sensitively react to the proton state (e.g., distance from the BAS). It was shown that upon protonation the T-O_B_ bond lengths are increased and Al-O_B_-Si angle is decreased.^8^ For our data set, the Al-O_B_ and Si-O_B_ are on average prolonged by 0.160±0.012 Å and 0.118±0.007 Å, respectively, and Al-O_B_-Si angle is on average decreased by 9.6 ± 7.4 degrees (Table S1). The large variance in change of Al-O_B_-Si angle most likely reflects the local flexibility of BAS. Additionally, a weak trend between these parameters is observed indicating importance of electronic structure effects at least for structures without intra-zeolite hydrogen bond (Figure S4). This observation is further supported by the fact that these geometric metrics do not correlate with proton confinement indicating possible correlation with deprotonation energy. However, the expected correlation was not found, further demonstrating that the relation of intrinsic acidity determined via deprotonation energy is not related to geometric changes of the BAS.

| **a** | **b** |
| --- | --- |
| 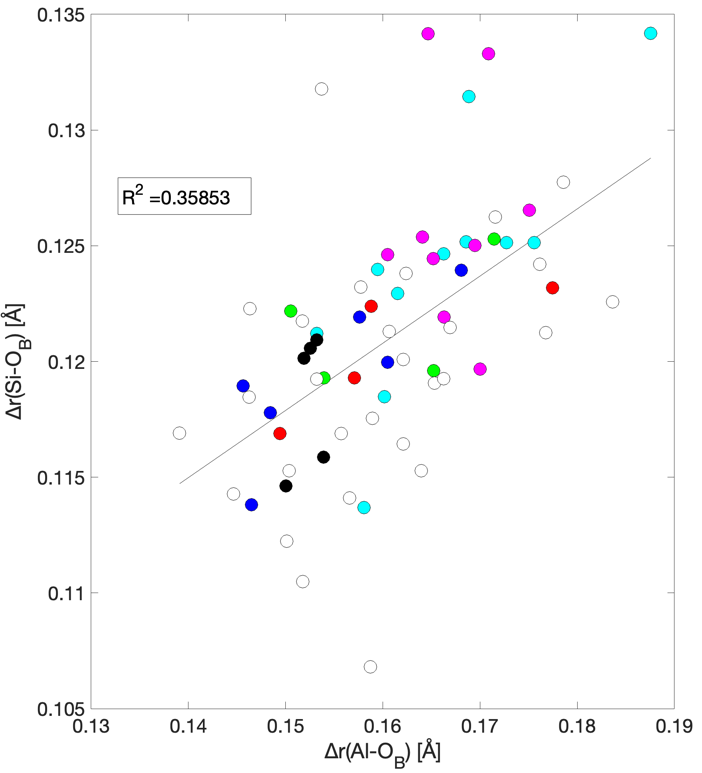 | 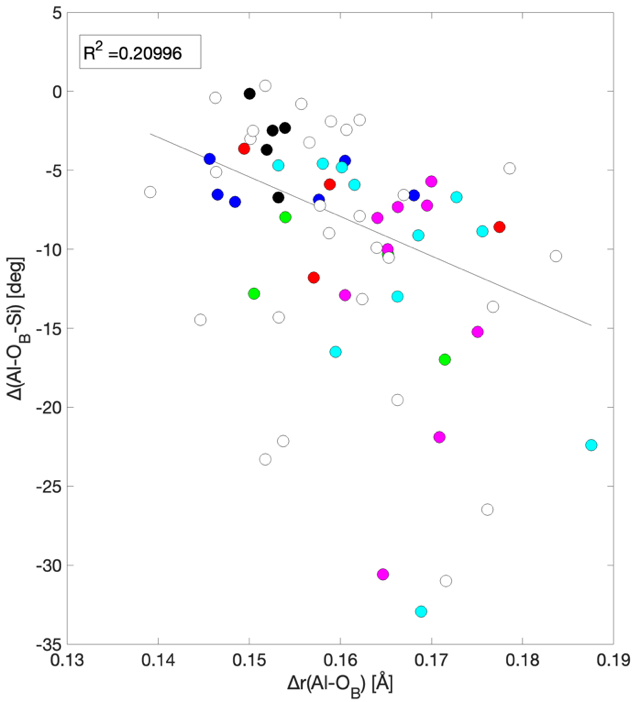 |
| **c** |  |
| 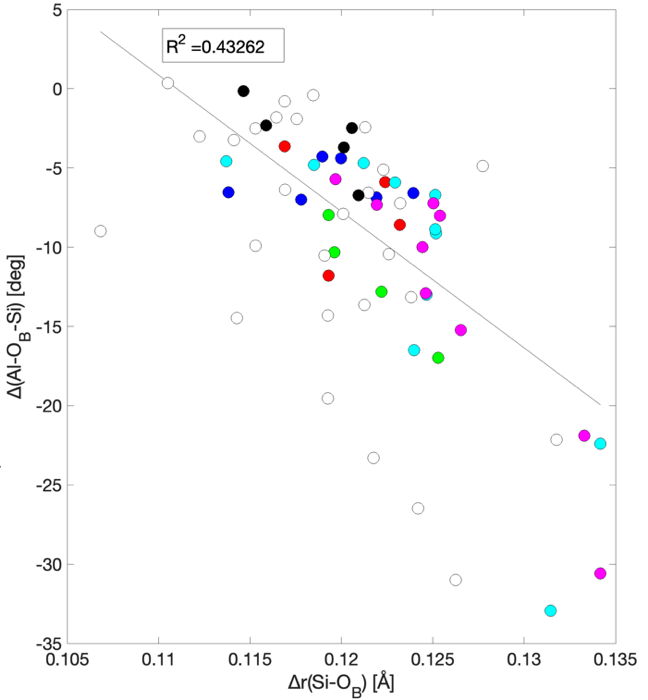 | 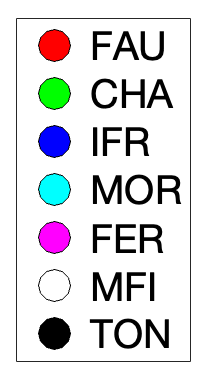 |
| **Figure S4** Correlation of changes in geometrical parameters between BAS and corresponding $\mathrm{AlO}_{4}^{-}$ anion for (a) ΔT-O_B_ bond lengths, (b) ΔAl-O_B_ bond length vs. ΔAl-O_B_-Si angle, and (c) ΔSi-O_B_ bond length vs. ΔAl-O_B_-Si angle. Note that BAS with intra-zeolitic hydrogen bond were excluded. | |

| **Table S1** Basic geometry parameters of BAS for the investigated aluminosilicates. The relative energies (E_rel_) are defined with respect to particular T-position of aluminosilicate framework. MFI structure was calculated only without the probe base adsorbates. | | | | | | | | | | | | | | | |
| --- | --- | --- | --- | --- | --- | --- | --- | --- | --- | --- | --- | --- | --- | --- | --- |
|  |  |  | **BAS** | | | | | | | | |  | **Anion** | | |
| Framework | T-site |  | E_rel_  [kJ.mol^-1^] | O_B_-H_B_  [Å] | Al-O_B_  [Å] | Si-O_B_  [Å] | Al-O_B_-Si  [deg] | H_B_-O_f_  [Å] | O_B_-H_B_-O_f_  [deg] | Al-O_B_-H_B_-Si  [deg] | η_O_  [Å^-6^] |  | Al-O_B_  [Å] | Si-O_B_  [Å] | Al-O_B_-Si  [deg] |
| FAU | Al1-O1 |  | 0.00 | 0.9754 | 1.8871 | 1.6951 | 127.1 | 2.58 | 83.9 | 173.1 | 0.0104 |  | 1.7300 | 1.5759 | 138.9 |
| FAU | Al1-O2 |  | 2.42 | 0.9795 | 1.9348 | 1.7132 | 127.4 | 2.66 | 134.3 | 173.8 | 0.0151 |  | 1.7574 | 1.5900 | 136.0 |
| FAU | Al1-O3 |  | 7.96 | 0.9810 | 1.8955 | 1.7014 | 131.0 | 2.60 | 136.5 | 176.0 | 0.0157 |  | 1.7367 | 1.5790 | 136.9 |
| FAU | Al1-O4 |  | 7.05 | 0.9765 | 1.8765 | 1.6901 | 130.4 | 2.54 | 79.5 | 170.8 | 0.0114 |  | 1.7271 | 1.5732 | 134.0 |
| CHA | Al1-O1 |  | 4.79 | 0.9767 | 1.9229 | 1.7115 | 131.5 | 2.63 | 78.4 | 177.3 | 0.0117 |  | 1.7514 | 1.5862 | 148.5 |
| CHA | Al1-O2 |  | 6.05 | 0.9779 | 1.8759 | 1.6959 | 133.8 | 2.45 | 90.2 | 165.2 | 0.0116 |  | 1.7253 | 1.5738 | 146.6 |
| CHA | Al1-O3 |  | 2.19 | 0.9779 | 1.8877 | 1.6964 | 134.9 | 2.46 | 89.8 | 168.1 | 0.0122 |  | 1.7337 | 1.5771 | 142.9 |
| CHA | Al1-O4 |  | 0.00 | 0.9761 | 1.9130 | 1.7037 | 130.9 | 2.58 | 85.9 | 172.8 | 0.0110 |  | 1.7478 | 1.5841 | 141.2 |
| IFR | Al1-O3 |  | 8.25 | 1.0091 | 1.8947 | 1.6840 | 135.4 | 1.74 | 156.4 | 153.6 | 0.0503 |  | 1.7329 | 1.5693 | 146.6 |
| IFR | Al1-O5 |  | 0.00 | 0.9947 | 1.9016 | 1.6908 | 123.6 | 1.94 | 176.6 | 172.0 | 0.0301 |  | 1.7498 | 1.5813 | 128.0 |
| IFR | Al1-O8 |  | 10.69 | 0.9751 | 1.8813 | 1.6957 | 127.3 | 2.58 | 76.9 | 173.9 | 0.0104 |  | 1.7329 | 1.5779 | 134.3 |
| IFR | Al1-O9 |  | 8.61 | 0.9834 | 1.9256 | 1.7049 | 131.0 | 2.35 | 140.6 | 169.4 | 0.0186 |  | 1.7511 | 1.5834 | 138.9 |
| IFR | Al2-O1 |  | 0.00 | 0.9934 | 1.8749 | 1.6981 | 136.0 | 1.89 | 146.2 | 171.0 | 0.0364 |  | 1.7322 | 1.5804 | 144.2 |
| IFR | Al2-O2 |  | 16.19 | 0.9946 | 1.9063 | 1.7040 | 127.3 | 1.93 | 178.6 | 160.4 | 0.0340 |  | 1.7611 | 1.5956 | 138.0 |
| IFR | Al2-O3 |  | 4.55 | 1.0016 | 1.8658 | 1.6943 | 134.5 | 1.81 | 148.5 | 152.5 | 0.0426 |  | 1.7185 | 1.5730 | 146.9 |
| IFR | Al2-O4 |  | 5.26 | 0.9774 | 1.8979 | 1.7005 | 134.2 | 2.56 | 86.2 | 167.0 | 0.0116 |  | 1.7374 | 1.5806 | 138.6 |
| IFR | Al3-O1 |  | 0.75 | 0.9971 | 1.8948 | 1.6871 | 134.1 | 1.86 | 150.6 | 167.4 | 0.0385 |  | 1.7407 | 1.5776 | 143.6 |
| IFR | Al3-O5 |  | 0.00 | 0.9902 | 1.8714 | 1.7011 | 125.4 | 2.06 | 168.5 | 170.5 | 0.0260 |  | 1.7381 | 1.5860 | 128.6 |
| IFR | Al3-O6 |  | 7.83 | 0.9794 | 1.9209 | 1.7137 | 128.9 | 2.62 | 79.2 | 172.2 | 0.0153 |  | 1.7529 | 1.5898 | 135.5 |
| IFR | Al3-O7 |  | 26.70 | 1.0028 | 1.8923 | 1.7113 | 132.2 | 1.82 | 159.0 | 159.5 | 0.0421 |  | 1.7449 | 1.5925 | 139.5 |
| IFR | Al4-O10 |  | 11.76 | 0.9775 | 1.8676 | 1.6922 | 134.2 | 2.48 | 81.3 | 177.1 | 0.0126 |  | 1.7220 | 1.5733 | 138.5 |
| IFR | Al4-O7 |  | 19.15 | 0.9925 | 1.9328 | 1.6991 | 133.4 | 1.95 | 150.2 | 159.1 | 0.0327 |  | 1.7576 | 1.5844 | 140.1 |
| IFR | Al4-O8 |  | 3.28 | 0.9755 | 1.8791 | 1.6925 | 126.3 | 2.53 | 85.7 | 166.0 | 0.0106 |  | 1.7326 | 1.5787 | 132.9 |
| IFR | Al4-O9 |  | 0.00 | 0.9833 | 1.9002 | 1.7086 | 129.1 | 2.45 | 138.9 | 167.1 | 0.0182 |  | 1.7426 | 1.5867 | 136.0 |
| MOR | Al1-O1 |  | 5.92 | 0.9777 | 1.9135 | 1.7060 | 135.8 | 2.52 | 89.4 | 175.6 | 0.0118 |  | 1.7449 | 1.5809 | 144.9 |
| MOR | Al1-O2 |  | 7.37 | 0.9778 | 1.9231 | 1.7064 | 137.0 | 2.49 | 90.4 | 178.4 | 0.0128 |  | 1.7476 | 1.5812 | 145.9 |
| MOR | Al1-O3 |  | 3.33 | 0.9762 | 1.9096 | 1.7090 | 128.4 | 2.60 | 79.6 | 173.7 | 0.0114 |  | 1.7408 | 1.5775 | 161.3 |
| MOR | Al1-O4 |  | 0.00 | 0.9780 | 1.9183 | 1.7091 | 134.0 | 2.51 | 90.0 | 165.3 | 0.0113 |  | 1.7456 | 1.5839 | 140.7 |
| MOR | Al2-O4 |  | 7.27 | 0.9773 | 1.9259 | 1.7124 | 137.0 | 2.49 | 83.2 | 177.0 | 0.0112 |  | 1.7384 | 1.5782 | 159.4 |
| MOR | Al2-O5 |  | 1.96 | 0.9773 | 1.9015 | 1.7018 | 135.4 | 2.54 | 88.4 | 179.0 | 0.0118 |  | 1.7352 | 1.5772 | 148.4 |
| MOR | Al2-O6 |  | 10.91 | 1.0204 | 1.9212 | 1.6969 | 147.6 | 1.62 | 169.2 | 172.5 | 0.0719 |  | 1.7475 | 1.5817 | 170.2 |
| MOR | Al2-O7 |  | 0.00 | 0.9766 | 1.8908 | 1.7028 | 132.7 | 2.52 | 81.6 | 167.7 | 0.0112 |  | 1.7376 | 1.5816 | 137.4 |
| MOR | Al3-O3 |  | 0.00 | 0.9759 | 1.9128 | 1.7074 | 128.0 | 2.74 | 82.4 | 178.1 | 0.0109 |  | 1.7526 | 1.5889 | 132.8 |
| MOR | Al3-O8 |  | 7.95 | 0.9783 | 1.8997 | 1.7009 | 136.2 | 2.51 | 89.1 | 168.9 | 0.0131 |  | 1.7382 | 1.5780 | 142.1 |
| MOR | Al3-O9 |  | 13.14 | 0.9982 | 1.9102 | 1.7034 | 141.6 | 1.79 | 155.9 | 173.7 | 0.0468 |  | 1.7481 | 1.5847 | 156.2 |
| MOR | Al4-O10 |  | 8.36 | 0.9787 | 1.8834 | 1.6937 | 135.2 | 2.45 | 90.9 | 161.7 | 0.0130 |  | 1.7239 | 1.5697 | 151.6 |
| MOR | Al4-O7 |  | 0.00 | 0.9760 | 1.9027 | 1.6945 | 129.8 | 2.59 | 85.4 | 171.0 | 0.0109 |  | 1.7446 | 1.5808 | 134.4 |
| MOR | Al4-O9 |  | 13.98 | 1.0058 | 1.9158 | 1.6922 | 138.5 | 1.71 | 161.9 | 171.7 | 0.0562 |  | 1.7545 | 1.5856 | 150.4 |
| FER | Al1-O1 |  | 4.43 | 0.9772 | 1.9098 | 1.7099 | 136.3 | 2.43 | 85.3 | 176.9 | 0.0126 |  | 1.7389 | 1.5766 | 158.2 |
| FER | Al1-O2 |  | 1.04 | 0.9778 | 1.8916 | 1.7103 | 130.0 | 2.62 | 78.7 | 178.4 | 0.0120 |  | 1.7269 | 1.5762 | 160.6 |
| FER | Al1-O3 |  | 0.00 | 0.9778 | 1.9015 | 1.7024 | 136.6 | 2.47 | 90.0 | 179.0 | 0.0126 |  | 1.7364 | 1.5780 | 146.6 |
| FER | Al1-O4 |  | 11.42 | 1.0097 | 1.9129 | 1.7022 | 147.9 | 1.71 | 163.9 | 171.6 | 0.0568 |  | 1.7465 | 1.5857 | 158.2 |
| FER | Al2-O2 |  | 0.00 | 0.9777 | 1.9173 | 1.7009 | 131.2 | 2.72 | 72.9 | 178.2 | 0.0109 |  | 1.7473 | 1.5813 | 136.9 |
| FER | Al2-O5 |  | 7.25 | 0.9789 | 1.9158 | 1.7017 | 137.7 | 2.48 | 83.1 | 172.5 | 0.0143 |  | 1.7407 | 1.5752 | 153.0 |
| FER | Al2-O6 |  | 18.50 | 0.9810 | 1.9046 | 1.6956 | 144.9 | 2.32 | 97.1 | 177.2 | 0.0152 |  | 1.7383 | 1.5737 | 152.3 |
| FER | Al3-O1 |  | 2.40 | 0.9766 | 1.9078 | 1.7089 | 131.2 | 2.57 | 87.4 | 168.8 | 0.0107 |  | 1.7438 | 1.5836 | 139.2 |
| FER | Al3-O7 |  | 0.00 | 1.0013 | 1.8969 | 1.7036 | 133.8 | 1.75 | 157.2 | 164.9 | 0.0484 |  | 1.7426 | 1.5820 | 157.7 |
| FER | Al3-O8 |  | 12.48 | 0.9781 | 1.9125 | 1.7060 | 138.1 | 2.45 | 91.6 | 176.2 | 0.0140 |  | 1.7430 | 1.5809 | 145.3 |
| FER | Al4-O5 |  | 2.17 | 0.9823 | 1.8998 | 1.7074 | 134.9 | 2.44 | 141.0 | 176.8 | 0.0171 |  | 1.7393 | 1.5827 | 147.8 |
| FER | Al4-O7 |  | 0.00 | 1.0017 | 1.9167 | 1.7002 | 133.0 | 1.79 | 158.7 | 163.1 | 0.0438 |  | 1.7492 | 1.5835 | 153.2 |
| MFI | Al1-O1 |  | 28.02 | 0.9789 | 1.8725 | 1.6927 | 139.6 | 2.44 | 81.9 | 174.7 | 0.0143 |  | 1.7334 | 1.5758 | 145.9 |
| MFI | Al1-O2 |  | 20.13 | 0.9756 | 1.9016 | 1.7000 | 122.1 | 2.63 | 84.3 | 152.3 | 0.0095 |  | 1.7451 | 1.5859 | 125.3 |
| MFI | Al1-O3 |  | 24.95 | 0.9762 | 1.9095 | 1.6971 | 132.7 | 2.55 | 80.1 | 173.1 | 0.0111 |  | 1.7334 | 1.5729 | 159.2 |
| MFI | Al1-O4 |  | 36.75 | 1.0070 | 1.9184 | 1.7009 | 144.3 | 1.75 | 160.2 | 161.2 | 0.0518 |  | 1.7624 | 1.5923 | 142.9 |
| MFI | Al2-O2 |  | 0.00 | 0.9743 | 1.8960 | 1.6953 | 125.0 | 2.65 | 82.8 | 179.5 | 0.0096 |  | 1.7459 | 1.5831 | 128.0 |
| MFI | Al2-O5 |  | 15.95 | 0.9765 | 1.8883 | 1.6953 | 132.0 | 2.55 | 86.8 | 168.9 | 0.0117 |  | 1.7379 | 1.5800 | 134.5 |
| MFI | Al2-O6 |  | 14.40 | 0.9792 | 1.9404 | 1.7082 | 138.9 | 2.60 | 87.0 | 169.9 | 0.0132 |  | 1.7567 | 1.5857 | 149.4 |
| MFI | Al2-O7 |  | 3.62 | 1.0128 | 1.8832 | 1.6873 | 131.4 | 1.63 | 158.4 | 174.6 | 0.0675 |  | 1.7484 | 1.5822 | 147.5 |
| MFI | Al3-O10 |  | 4.02 | 1.0103 | 1.9249 | 1.6998 | 142.0 | 1.72 | 160.3 | 169.4 | 0.0525 |  | 1.7470 | 1.5842 | 141.5 |
| MFI | Al3-O5 |  | 8.09 | 0.9770 | 1.8743 | 1.6989 | 134.3 | 2.50 | 88.6 | 171.6 | 0.0123 |  | 1.7280 | 1.5766 | 139.4 |
| MFI | Al3-O8 |  | 2.28 | 0.9764 | 1.9001 | 1.6963 | 131.6 | 2.52 | 88.1 | 173.8 | 0.0108 |  | 1.7348 | 1.5773 | 142.1 |
| MFI | Al3-O9 |  | 0.00 | 1.0224 | 1.8826 | 1.6805 | 139.0 | 1.57 | 164.4 | 178.8 | 0.0799 |  | 1.7506 | 1.5826 | 152.1 |
| MFI | Al4-O11 |  | 12.02 | 0.9809 | 1.9101 | 1.7122 | 134.6 | 2.48 | 91.3 | 159.0 | 0.0151 |  | 1.7494 | 1.5909 | 137.0 |
| MFI | Al4-O12 |  | 16.61 | 0.9798 | 1.8987 | 1.7040 | 136.8 | 2.33 | 94.6 | 168.8 | 0.0151 |  | 1.7429 | 1.5871 | 137.6 |
| MFI | Al4-O4 |  | 0.00 | 1.0177 | 1.9020 | 1.6921 | 136.9 | 1.61 | 164.5 | 172.7 | 0.0717 |  | 1.7466 | 1.5855 | 150.7 |
| MFI | Al4-O9 |  | 22.98 | 0.9967 | 1.8884 | 1.7048 | 137.5 | 1.88 | 152.3 | 164.7 | 0.0368 |  | 1.7409 | 1.5826 | 158.4 |
| MFI | Al5-O1 |  | 13.23 | 0.9754 | 1.8855 | 1.6931 | 126.7 | 2.66 | 83.1 | 169.8 | 0.0102 |  | 1.7409 | 1.5788 | 141.2 |
| MFI | Al5-O12 |  | 0.00 | 0.9939 | 1.9087 | 1.6966 | 132.5 | 1.93 | 152.8 | 174.2 | 0.0321 |  | 1.7510 | 1.5843 | 140.3 |
| MFI | Al5-O13 |  | 7.97 | 0.9778 | 1.8869 | 1.6991 | 137.2 | 2.47 | 82.4 | 169.4 | 0.0125 |  | 1.7291 | 1.5759 | 144.4 |
| MFI | Al5-O14 |  | 9.32 | 1.0161 | 1.8964 | 1.6896 | 138.8 | 1.65 | 163.6 | 173.0 | 0.0642 |  | 1.7492 | 1.5842 | 145.8 |
| MFI | Al6-O10 |  | 27.42 | 1.0073 | 1.8999 | 1.6904 | 136.8 | 1.72 | 162.4 | 179.5 | 0.0521 |  | 1.7476 | 1.5838 | 154.3 |
| MFI | Al6-O13 |  | 0.00 | 0.9765 | 1.8984 | 1.6923 | 132.8 | 2.45 | 90.1 | 173.3 | 0.0116 |  | 1.7467 | 1.5818 | 132.4 |
| MFI | Al6-O15 |  | 3.10 | 0.9772 | 1.8865 | 1.6944 | 131.0 | 2.50 | 87.4 | 160.1 | 0.0119 |  | 1.7348 | 1.5726 | 154.3 |
| MFI | Al6-O7 |  | 2.22 | 0.9916 | 1.9164 | 1.6991 | 129.6 | 1.98 | 150.0 | 162.0 | 0.0316 |  | 1.7389 | 1.5807 | 157.2 |
| MFI | Al7-O11 |  | 11.32 | 0.9777 | 1.9305 | 1.7016 | 134.8 | 2.46 | 90.8 | 175.7 | 0.0124 |  | 1.7538 | 1.5804 | 148.4 |
| MFI | Al7-O16 |  | 8.72 | 0.9787 | 1.8993 | 1.6856 | 135.5 | 2.28 | 94.7 | 179.2 | 0.0154 |  | 1.7406 | 1.5788 | 144.5 |
| MFI | Al7-O17 |  | 0.00 | 0.9750 | 1.9107 | 1.7037 | 125.7 | 2.57 | 79.9 | 167.7 | 0.0098 |  | 1.7518 | 1.5861 | 127.6 |
| MFI | Al7-O18 |  | 19.27 | 0.9799 | 1.9012 | 1.6969 | 141.8 | 2.36 | 94.5 | 175.3 | 0.0165 |  | 1.7296 | 1.5707 | 172.8 |
| MFI | Al8-O17 |  | 0.00 | 1.0039 | 1.8713 | 1.6867 | 135.9 | 1.72 | 157.6 | 178.0 | 0.0522 |  | 1.7332 | 1.5767 | 144.7 |
| MFI | Al8-O19 |  | 23.69 | 0.9821 | 1.8990 | 1.7005 | 139.9 | 2.20 | 99.4 | 164.4 | 0.0171 |  | 1.7321 | 1.5791 | 146.4 |
| MFI | Al8-O20 |  | 19.04 | 1.0123 | 1.9225 | 1.6935 | 142.4 | 1.69 | 167.2 | 162.5 | 0.0588 |  | 1.7514 | 1.5907 | 146.0 |
| MFI | Al8-O6 |  | 8.92 | 0.9993 | 1.8978 | 1.6973 | 136.9 | 1.83 | 152.9 | 165.4 | 0.0410 |  | 1.7437 | 1.5839 | 147.6 |
| MFI | Al9-O15 |  | 2.34 | 0.9762 | 1.8924 | 1.6936 | 123.9 | 2.46 | 88.9 | 155.5 | 0.0111 |  | 1.7262 | 1.5743 | 143.5 |
| MFI | Al9-O19 |  | 1.74 | 0.9829 | 1.9202 | 1.7066 | 136.2 | 2.44 | 140.2 | 164.6 | 0.0169 |  | 1.7416 | 1.5788 | 141.0 |
| MFI | Al9-O21 |  | 0.00 | 0.9767 | 1.8807 | 1.6916 | 133.2 | 2.46 | 88.4 | 178.9 | 0.0125 |  | 1.7275 | 1.5724 | 147.5 |
| MFI | Al9-O22 |  | 22.65 | 0.9966 | 1.9495 | 1.7033 | 138.8 | 1.89 | 145.6 | 163.2 | 0.0370 |  | 1.7544 | 1.5891 | 139.8 |
| MFI | Al10-O22 |  | 16.31 | 1.0010 | 1.9022 | 1.7011 | 137.8 | 1.76 | 156.1 | 168.3 | 0.0499 |  | 1.7572 | 1.5910 | 138.4 |
| MFI | Al10-O23 |  | 7.63 | 0.9750 | 1.9025 | 1.6955 | 125.6 | 2.60 | 84.5 | 167.9 | 0.0107 |  | 1.7386 | 1.5802 | 135.5 |
| MFI | Al10-O24 |  | 0.00 | 1.0090 | 1.8954 | 1.6920 | 136.1 | 1.72 | 163.5 | 177.9 | 0.0528 |  | 1.7442 | 1.5846 | 143.8 |
| MFI | Al10-O3 |  | 5.97 | 0.9765 | 1.8839 | 1.7025 | 131.2 | 2.55 | 87.1 | 179.2 | 0.0107 |  | 1.7377 | 1.5841 | 131.6 |
| MFI | Al11-O14 |  | 22.33 | 1.0049 | 1.9032 | 1.6976 | 137.0 | 1.72 | 157.2 | 167.1 | 0.0547 |  | 1.7403 | 1.5847 | 144.1 |
| MFI | Al11-O16 |  | 33.69 | 0.9794 | 1.9059 | 1.7000 | 137.5 | 2.36 | 94.0 | 163.8 | 0.0142 |  | 1.7438 | 1.5836 | 139.3 |
| MFI | Al11-O24 |  | 0.00 | 1.0028 | 1.8963 | 1.6940 | 136.9 | 1.75 | 157.5 | 179.7 | 0.0500 |  | 1.7444 | 1.5818 | 144.0 |
| MFI | Al11-O25 |  | 4.39 | 0.9870 | 1.8980 | 1.7046 | 137.0 | 2.12 | 150.0 | 176.9 | 0.0247 |  | 1.7480 | 1.5880 | 132.2 |
| MFI | Al12-O20 |  | 3.03 | 1.0035 | 1.9058 | 1.6993 | 144.9 | 1.77 | 157.4 | 173.0 | 0.0492 |  | 1.7404 | 1.5803 | 162.5 |
| MFI | Al12-O25 |  | 2.56 | 0.9749 | 1.9084 | 1.7038 | 128.9 | 2.60 | 79.9 | 176.5 | 0.0100 |  | 1.7460 | 1.5800 | 142.0 |
| MFI | Al12-O26 |  | 0.00 | 0.9797 | 1.8948 | 1.6937 | 139.5 | 2.33 | 94.4 | 170.7 | 0.0150 |  | 1.7327 | 1.5736 | 147.4 |
| MFI | Al12-O8 |  | 2.97 | 0.9754 | 1.8881 | 1.7071 | 130.3 | 2.54 | 80.8 | 179.5 | 0.0102 |  | 1.7344 | 1.5753 | 152.4 |
| TON | Al1-O1 |  | 9.15 | 0.9792 | 1.9034 | 1.7022 | 128.4 | 2.49 | 141.7 | 171.4 | 0.0157 |  | 1.7533 | 1.5876 | 128.6 |
| TON | Al1-O2 |  | 0.00 | 1.0109 | 1.9303 | 1.6915 | 140.7 | 1.72 | 161.6 | 173.5 | 0.0535 |  | 1.7484 | 1.5783 | 152.7 |
| TON | Al1-O3 |  | 6.27 | 0.9765 | 1.8954 | 1.7036 | 131.8 | 2.72 | 72.1 | 177.6 | 0.0103 |  | 1.7428 | 1.5831 | 134.3 |
| TON | Al2-O3 |  | 0.00 | 0.9775 | 1.8951 | 1.6996 | 130.0 | 2.59 | 85.7 | 156.9 | 0.0112 |  | 1.7412 | 1.5838 | 132.3 |
| TON | Al2-O4 |  | 14.80 | 0.9789 | 1.8846 | 1.6994 | 137.6 | 2.43 | 91.8 | 171.5 | 0.0134 |  | 1.7314 | 1.5785 | 144.3 |
| TON | Al2-O5 |  | 5.63 | 1.0002 | 1.9021 | 1.6984 | 133.2 | 1.83 | 161.5 | 166.9 | 0.0410 |  | 1.7579 | 1.5889 | 136.7 |
| TON | Al3-O4 |  | 0.00 | 0.9769 | 1.8855 | 1.7009 | 134.0 | 2.50 | 88.7 | 174.9 | 0.0122 |  | 1.7336 | 1.5807 | 137.7 |
| TON | Al3-O6a |  | 18.30 | 0.9820 | 1.9267 | 1.7092 | 133.0 | 2.38 | 142.0 | 170.6 | 0.0197 |  | 1.7538 | 1.5850 | 139.8 |
| TON | Al3-O6b |  | 21.41 | 0.9830 | 1.9010 | 1.7078 | 133.0 | 2.30 | 140.3 | 179.5 | 0.0210 |  | 1.7489 | 1.5903 | 133.5 |
| TON | Al4-O2 |  | 0.00 | 0.9988 | 1.8896 | 1.7079 | 132.2 | 1.81 | 153.8 | 159.9 | 0.0436 |  | 1.7418 | 1.5886 | 138.2 |
| TON | Al4-O6a |  | 10.51 | 0.9823 | 1.9112 | 1.7140 | 131.8 | 2.29 | 142.0 | 174.6 | 0.0210 |  | 1.7537 | 1.5882 | 134.2 |
| TON | Al4-O6b |  | 3.47 | 0.9833 | 1.9224 | 1.7032 | 135.2 | 2.33 | 143.5 | 177.8 | 0.0194 |  | 1.7464 | 1.5894 | 141.0 |

**Table S2** Thermodynamically the most stable **ammonia** adsorption complexes for investigated aluminosilicate’s frameworks at different T-positions along with adsorption complexes formed at the most stable BAS.

| **Framework** | **T-site** | **E_rel_ (BAS)**  **[kJ.mol^-1^]** | **PBE**  **[kJ.mol^-1^]** | **D2**  **[kJ.mol^-1^]** | **PBE-D2**  **[kJ.mol^-1^]** | **O_B_-H_B_**  **[Å]** | **H_B_-N**  **[Å]** | **O_B_-N**  **[Å]** | **O_B_-H_B_-N**  **[deg]** | **Al-O_B_**  **[Å]** | **Si-O_B_**  **[Å]** | **Al-O_B_-Si**  **[deg]** | **Al-O_B_-H_B_-Si**  **[deg]** | **n(Al,Si)^b^** |
| --- | --- | --- | --- | --- | --- | --- | --- | --- | --- | --- | --- | --- | --- | --- |
| H-FAU | Al1-O1 | 0.0 | -127.1 | -15.8 | -143.0 | 1.6407 | 1.0761 | 2.6347 | 151.1 | 1.7775 | 1.6156 | 130.0 | 178.8 | (2,0) |
| H-CHA | Al1-O4 | 0.0 | -136.2 | -26.6 | -162.8 | 1.5814 | 1.0893 | 2.6704 | 178.1 | 1.7979 | 1.6207 | 130.6 | 176.0 | (1,1) |
| H-IFR | Al1-O8^a^ | 10.7 | -123.1 | -20.7 | -143.8 | 1.5476 | 1.0995 | 2.6445 | 174.8 | 1.7864 | 1.6192 | 127.5 | 160.2 | (1,1) |
| H-IFR | Al2-O4 | 5.3 | -127.0 | -20.8 | -147.8 | 1.5770 | 1.0950 | 2.6676 | 173.3 | 1.7871 | 1.6192 | 131.1 | 166.7 | (1,1) |
| H-IFR | Al2-O1 | 0.0 | -117.0 | -23.6 | -140.6 | 1.6987 | 1.0673 | 2.6879 | 151.9 | 1.7678 | 1.6112 | 138.2 | 164.4 | (2,0) |
| H-IFR | Al3-O6 | 7.8 | -120.1 | -26.5 | -146.5 | 1.5487 | 1.0945 | 2.6297 | 168.2 | 1.8002 | 1.6253 | 132.3 | 170.1 | (1,1) |
| H-IFR | Al3-O5 | 0.0 | -104.2 | -29.6 | -133.8 | 1.7249 | 1.0677 | 2.7185 | 152.8 | 1.7693 | 1.6148 | 127.7 | 141.7 | (2,0) |
| H-IFR | Al4-O8 | 3.3 | -125.4 | -17.6 | -143.0 | 1.6052 | 1.0830 | 2.6254 | 154.7 | 1.7790 | 1.6158 | 126.8 | 171.3 | (2,0) |
| H-IFR | Al4-O9 | 0.0 | -115.5 | -27.6 | -143.0 | 1.5627 | 1.0917 | 2.6403 | 168.0 | 1.7879 | 1.6213 | 133.0 | 169.8 | (1,1) |
| H-MOR | Al1-O2 | 7.4 | -147.6 | -35.1 | -182.6 | 1.6864 | 1.0710 | 2.7484 | 170.5 | 1.7904 | 1.6141 | 137.0 | 169.8 | (1,2) |
| H-MOR | Al1-O4 | 0.0 | -121.9 | -20.1 | -142.0 | 1.6727 | 1.0727 | 2.6594 | 150.5 | 1.7911 | 1.6221 | 133.4 | 175.2 | (2,0) |
| H-MOR | Al2-O5^a^ | 2.0 | -135.3 | -23.5 | -158.8 | 1.7474 | 1.0622 | 2.7126 | 148.8 | 1.7732 | 1.6096 | 138.2 | 178.7 | (2,0) |
| H-MOR | Al3-O3 | 0.0 | -135.3 | -33.1 | -168.4 | 1.5866 | 1.0831 | 2.6684 | 176.3 | 1.7939 | 1.6195 | 131.7 | 164.8 | (1,2) |
| H-MOR | Al4-O7 | 0.0 | -132.2 | -23.3 | -155.6 | 1.5383 | 1.0965 | 2.6334 | 176.3 | 1.7916 | 1.6154 | 131.5 | 174.5 | (1,1) |
| H-FER | Al1-O2 | 1.0 | -132.3 | -28.4 | -160.7 | 1.7048 | 1.0652 | 2.7011 | 153.7 | 1.7802 | 1.6189 | 130.7 | 161.8 | (2,1) |
| H-FER | Al1-O3 | 0.0 | -122.3 | -30.3 | -152.6 | 1.5693 | 1.0923 | 2.6578 | 173.7 | 1.7885 | 1.6151 | 138.6 | 164.1 | (1,1) |
| H-FER | Al2-O6 | 18.5 | -145.7 | -28.3 | -174.0 | 1.6853 | 1.0722 | 2.7571 | 178.0 | 1.7820 | 1.6076 | 143.6 | 177.8 | (1,1) |
| H-FER | Al2-O2 | 0.0 | -117.4 | -23.3 | -140.7 | 1.5254 | 1.1032 | 2.6236 | 172.8 | 1.8031 | 1.6208 | 132.6 | 163.5 | (1,1) |
| H-FER | Al3-O1 | 2.4 | -123.5 | -26.0 | -149.5 | 1.5610 | 1.0947 | 2.6493 | 171.9 | 1.7950 | 1.6229 | 131.8 | 160.8 | (1,1) |
| H-FER | Al3-O7 | 0.0 | -115.7 | -30.3 | -146.1 | 1.7531 | 1.0597 | 2.7121 | 148.2 | 1.7845 | 1.6193 | 135.3 | 175.9 | (2,0) |
| H-FER | Al4-O5^a^ | 2.2 | -119.6 | -30.8 | -150.4 | 1.6602 | 1.0775 | 2.7324 | 172.7 | 1.7850 | 1.6195 | 137.9 | 160.7 | (1,1) |
| H-TON | Al1-O3^a^ | 6.3 | -130.0 | -26.0 | -156.0 | 1.5675 | 1.0928 | 2.6458 | 167.8 | 1.7889 | 1.6198 | 129.8 | 169.0 | (1,1) |
| H-TON | Al2-O3 | 0.0 | -134.1 | -23.0 | -157.1 | 1.5972 | 1.0896 | 2.6849 | 175.6 | 1.7869 | 1.6185 | 127.8 | 154.8 | (1,1) |
| H-TON | Al3-O4 | 0.0 | -129.3 | -24.5 | -153.7 | 1.5768 | 1.0947 | 2.6704 | 176.5 | 1.7854 | 1.6201 | 130.2 | 174.4 | (1,1) |
| H-TON | Al4-O6b^a^ | 3.5 | -111.4 | -31.6 | -143.0 | 1.6555 | 1.0831 | 2.7310 | 171.3 | 1.8005 | 1.6172 | 132.1 | 172.2 | (1,0) |

^a^ The most stable BAS is either inaccessible to NH_3_ or upon adsorption the protons are re-shuffled that the closest BAS oxygen is changed.

^b^ Number of hydrogen bonds that ammonia forms with oxygens connected to Al and Si framework atoms.

**Table S3** Thermodynamically the most stable **formamide** adsorption complexes for investigated aluminosilicate’s frameworks at different T-positions along with adsorption complexes formed at the most stable BAS if accessible.

| **Framework** | **T-site** | **E_rel_ (BAS)**  **[kJ.mol^-1^]** | **PBE**  **[kJ.mol^-1^]** | **D2**  **[kJ.mol^-1^]** | **PBE-D2**  **[kJ.mol^-1^]** | **O_B_-H_B_**  **[Å]** | **H_B_-O**  **[Å]** | **O_B_-O**  **[Å]** | **O_B_-H_B_-O**  **[deg]** | **O-C**  **[Å]** | **H_B_-O-C**  **[deg]** | **Al-O_B_**  **[Å]** | **Si-O_B_**  **[Å]** | **Al-O_B_-Si**  **[deg]** | **Al-O_B_-H_B_-Si**  **[deg]** |
| --- | --- | --- | --- | --- | --- | --- | --- | --- | --- | --- | --- | --- | --- | --- | --- |
| H-FAU | Al1-O1 | 0.0 | -112.8 | -26.0 | -138.9 | 1.3483 | 1.1189 | 2.4637 | 173.9 | 1.2770 | 118.8 | 1.7987 | 1.6408 | 123.4 | 170.6 |
| H-CHA | Al1-O2 | 6.0 | -112.8 | -41.2 | -154.0 | 1.4202 | 1.0847 | 2.4941 | 169.2 | 1.2866 | 114.0 | 1.7835 | 1.6303 | 131.7 | 174.9 |
| H-CHA | Al1-O4 | 0.0 | -98.5 | -38.2 | -136.7 | 1.2809 | 1.1559 | 2.4348 | 175.4 | 1.2720 | 112.4 | 1.8254 | 1.6436 | 132.3 | 164.7 |
| H-IFR | Al1-O8 | 10.7 | -110.0 | -36.9 | -146.9 | 1.3688 | 1.1078 | 2.4709 | 172.2 | 1.2781 | 118.5 | 1.7944 | 1.6352 | 124.7 | 166.2 |
| H-IFR | Al1-O5 | 0.0 | -83.5 | -34.4 | -118.0 | 1.4230 | 1.0917 | 2.4984 | 166.9 | 1.2815 | 120.6 | 1.8017 | 1.6305 | 125.7 | 141.9 |
| H-IFR | Al2-O3 | 4.6 | -111.4 | -34.0 | -145.3 | 1.4460 | 1.0763 | 2.5208 | 175.9 | 1.2825 | 118.1 | 1.7735 | 1.6270 | 135.2 | 171.0 |
| H-IFR | Al2-O1 | 0.0 | -99.1 | -32.3 | -131.4 | 1.3501 | 1.1219 | 2.4634 | 170.4 | 1.2777 | 119.7 | 1.7958 | 1.6411 | 129.4 | 164.0 |
| H-IFR | Al3-O1 | 0.8 | -85.3 | -41.9 | -127.2 | 1.3295 | 1.1318 | 2.4533 | 170.7 | 1.2752 | 119.6 | 1.8045 | 1.6363 | 128.2 | 160.7 |
| H-IFR | Al3-O5 | 0.0 | -83.4 | -40.0 | -123.5 | 1.3886 | 1.1024 | 2.4884 | 174.7 | 1.2801 | 116.2 | 1.7953 | 1.6384 | 126.8 | 145.1 |
| H-IFR | Al4-O8 | 3.3 | -109.5 | -31.7 | -141.2 | 1.3510 | 1.1169 | 2.4639 | 173.4 | 1.2781 | 118.2 | 1.7999 | 1.6384 | 122.7 | 170.8 |
| H-IFR | Al4-O9 | 0.0 | -99.2 | -36.6 | -135.7 | 1.3822 | 1.1032 | 2.4843 | 176.6 | 1.2784 | 119.5 | 1.7997 | 1.6410 | 130.8 | 163.0 |
| H-MOR | Al1-O4 | 0.0 | -107.2 | -36.0 | -143.2 | 1.3775 | 1.1066 | 2.4761 | 170.7 | 1.2802 | 117.8 | 1.8138 | 1.6452 | 129.4 | 177.6 |
| H-MOR | Al2-O7 | 0.0 | -114.2 | -39.6 | -153.9 | 1.4040 | 1.0893 | 2.4888 | 173.1 | 1.2826 | 114.4 | 1.7957 | 1.6332 | 130.8 | 175.5 |
| H-MOR | Al3-O3 | 0.0 | -94.0 | -55.2 | -149.3 | 1.3692 | 1.1002 | 2.4678 | 175.9 | 1.2769 | 115.7 | 1.8132 | 1.6412 | 127.8 | 153.6 |
| H-MOR | Al4-O10 | 8.4 | -114.7 | -30.8 | -145.5 | 1.3693 | 1.1100 | 2.4745 | 172.9 | 1.2786 | 119.4 | 1.7911 | 1.6323 | 132.7 | 176.3 |
| H-MOR | Al4-O7 | 0.0 | -94.6 | -30.7 | -125.3 | 1.1714 | 1.2607 | 2.4289 | 174.1 | 1.2602 | 118.4 | 1.8362 | 1.6505 | 126.6 | 161.2 |
| H-FER | Al1-O2 | 1.0 | -101.0 | -42.8 | -143.8 | 1.3551 | 1.1150 | 2.4647 | 172.4 | 1.2781 | 121.6 | 1.8011 | 1.6456 | 128.6 | 172.4 |
| H-FER | Al1-O3 | 0.0 | -93.0 | -43.1 | -136.2 | 1.2292 | 1.2005 | 2.4289 | 177.1 | 1.2650 | 115.1 | 1.8312 | 1.6480 | 133.2 | 173.1 |
| H-FER | Al2-O5 | 7.2 | -106.3 | -44.3 | -150.5 | 1.3790 | 1.1065 | 2.4808 | 172.9 | 1.2803 | 120.0 | 1.8059 | 1.6345 | 138.3 | 175.1 |
| H-FER | Al2-O2 | 0.0 | -90.7 | -43.5 | -134.2 | 1.2455 | 1.1869 | 2.4321 | 178.2 | 1.2676 | 116.1 | 1.8372 | 1.6441 | 129.2 | 168.1 |
| H-FER | Al3-O1 | 2.4 | -103.4 | -40.3 | -143.7 | 1.2124 | 1.2060 | 2.4161 | 174.9 | 1.2660 | 115.6 | 1.8286 | 1.6506 | 129.4 | 177.0 |
| H-FER | Al3-O7 | 0.0 | -86.6 | -44.0 | -130.6 | 1.1824 | 1.2483 | 2.4242 | 171.6 | 1.2632 | 116.9 | 1.8378 | 1.6619 | 131.5 | 179.0 |
| H-FER | Al4-O7 | 0.0 | -91.6 | -43.7 | -135.3 | 1.3394 | 1.1274 | 2.4642 | 174.7 | 1.2750 | 119.9 | 1.8222 | 1.6464 | 134.6 | 178.6 |
| H-TON | Al1-O1 | 9.1 | -120.8 | -42.4 | -163.2 | 1.4559 | 1.0739 | 2.5273 | 174.9 | 1.2851 | 118.8 | 1.7994 | 1.6331 | 125.8 | 153.0 |
| H-TON | Al2-O3 | 0.0 | -113.5 | -44.1 | -157.6 | 1.4003 | 1.0905 | 2.4848 | 172.0 | 1.2829 | 115.6 | 1.7988 | 1.6344 | 127.2 | 170.0 |
| H-TON | Al3-O4 | 0.0 | -103.9 | -41.4 | -145.3 | 1.2471 | 1.1833 | 2.4260 | 173.1 | 1.2673 | 116.8 | 1.8130 | 1.6451 | 130.0 | 172.4 |
| H-TON | Al4-O6b | 3.5 | -77.4 | -47.7 | -125.2 | 1.1336 | 1.3199 | 2.4445 | 170.2 | 1.2572 | 123.3 | 1.8573 | 1.6623 | 132.1 | 169.6 |

**Table S4** Thermodynamically the most stable **acetone** adsorption complexes for investigated aluminosilicate’s frameworks at different T-positions along with adsorption complexes formed at the most stable BAS if accessible.

| **Framework** | **T-site** | **E_rel_ (BAS)**  **[kJ.mol^-1^]** | **PBE**  **[kJ.mol^-1^]** | **D2**  **[kJ.mol^-1^]** | **PBE-D2**  **[kJ.mol^-1^]** | **O_B_-H_B_**  **[Å]** | **H_B_-O**  **[Å]** | **O_B_-O**  **[Å]** | **O_B_-H_B_-O**  **[deg]** | **O-C**  **[Å]** | **H_B_-O-C**  **[deg]** | **Al-O_B_**  **[Å]** | **Si-O_B_**  **[Å]** | **Al-O_B_-Si**  **[deg]** | **Al-O_B_-H_B_-Si**  **[deg]** |
| --- | --- | --- | --- | --- | --- | --- | --- | --- | --- | --- | --- | --- | --- | --- | --- |
| H-FAU | Al1-O1 | 0.0 | -87.2 | -27.0 | -114.3 | 1.1282 | 1.3198 | 2.4466 | 176.1 | 1.2493 | 120.8 | 1.8346 | 1.6552 | 125.6 | 171.5 |
| H-CHA | Al1-O2 | 6.0 | -85.9 | -40.0 | -125.9 | 1.1235 | 1.3329 | 2.4538 | 174.7 | 1.2508 | 122.7 | 1.8284 | 1.6553 | 132.0 | 173.4 |
| H-CHA | Al1-O4 | 0.0 | -70.6 | -51.0 | -121.6 | 1.0981 | 1.3799 | 2.4737 | 173.2 | 1.2480 | 127.9 | 1.8620 | 1.6684 | 131.2 | 171.6 |
| H-IFR | Al1-O8 | 10.7 | -87.5 | -37.4 | -124.9 | 1.1304 | 1.3163 | 2.4460 | 177.0 | 1.2498 | 121.7 | 1.8290 | 1.6547 | 125.1 | 174.5 |
| H-IFR | Al1-O5 | 0.0 | -60.1 | -48.3 | -108.4 | 1.1263 | 1.3383 | 2.4636 | 176.7 | 1.2507 | 125.8 | 1.8540 | 1.6565 | 126.1 | 144.2 |
| H-IFR | Al2-O4 | 5.3 | -90.8 | -40.3 | -131.1 | 1.1110 | 1.3534 | 2.4585 | 172.0 | 1.2489 | 127.0 | 1.8393 | 1.6616 | 131.3 | 171.4 |
| H-IFR | Al2-O1 | 0.0 | -76.9 | -40.5 | -117.4 | 1.1214 | 1.3362 | 2.4552 | 174.9 | 1.2502 | 124.4 | 1.8303 | 1.6604 | 129.0 | 169.4 |
| H-IFR | Al3-O1 | 0.8 | -70.2 | -42.6 | -112.7 | 1.0998 | 1.3771 | 2.4730 | 173.6 | 1.2478 | 126.7 | 1.8493 | 1.6611 | 127.4 | 171.7 |
| H-IFR | Al3-O5 | 0.0 | -62.2 | -48.0 | -110.2 | 1.1219 | 1.3439 | 2.4641 | 175.7 | 1.2508 | 125.8 | 1.8368 | 1.6653 | 127.1 | 145.9 |
| H-IFR | Al4-O8 | 3.3 | -86.5 | -36.5 | -123.0 | 1.1129 | 1.3464 | 2.4548 | 173.1 | 1.2483 | 123.6 | 1.8328 | 1.6558 | 123.9 | 158.7 |
| H-IFR | Al4-O9 | 0.0 | -73.7 | -43.1 | -116.8 | 1.0882 | 1.4067 | 2.4909 | 173.5 | 1.2480 | 131.7 | 1.8530 | 1.6766 | 129.3 | 160.3 |
| H-MOR | Al1-O4 | 0.0 | -86.7 | -41.8 | -128.5 | 1.1111 | 1.3528 | 2.4606 | 174.0 | 1.2493 | 123.7 | 1.8582 | 1.6677 | 131.5 | 179.6 |
| H-MOR | Al2-O7 | 0.0 | -86.8 | -37.4 | -124.2 | 1.1020 | 1.3696 | 2.4669 | 172.9 | 1.2478 | 126.4 | 1.8449 | 1.6662 | 129.7 | 169.2 |
| H-MOR | Al3-O3 | 0.0 | -22.0 | -91.2 | -113.2 | 1.1100 | 1.3654 | 2.4607 | 167.4 | 1.2511 | 125.1 | 1.8695 | 1.6705 | 129.6 | 142.2 |
| H-MOR | Al4-O7 | 0.0 | -82.3 | -38.9 | -121.1 | 1.1098 | 1.3581 | 2.4639 | 173.4 | 1.2494 | 124.4 | 1.8511 | 1.6600 | 127.0 | 161.0 |
| H-FER | Al1-O2 | 1.0 | -76.1 | -63.2 | -139.3 | 1.1549 | 1.2852 | 2.4333 | 171.5 | 1.2530 | 121.3 | 1.8396 | 1.6654 | 129.3 | 158.0 |
| H-FER | Al1-O3 | 0.0 | -69.1 | -66.7 | -135.9 | 1.1170 | 1.3493 | 2.4622 | 173.4 | 1.2483 | 126.1 | 1.8532 | 1.6642 | 131.6 | 174.3 |
| H-FER | Al2-O2 | 0.0 | -72.0 | -56.9 | -128.9 | 1.0893 | 1.3964 | 2.4614 | 163.8 | 1.2481 | 123.3 | 1.8657 | 1.6671 | 130.5 | 178.1 |
| H-FER | Al3-O1 | 2.4 | -70.2 | -62.8 | -133.1 | 1.0926 | 1.3726 | 2.4531 | 168.5 | 1.2473 | 126.1 | 1.8631 | 1.6675 | 127.9 | 177.6 |
| H-FER | Al3-O7 | 0.0 | -64.2 | -62.4 | -126.6 | 1.0753 | 1.4277 | 2.4897 | 168.0 | 1.2459 | 130.1 | 1.8630 | 1.6787 | 131.9 | 174.5 |
| H-FER | Al4-O7 | 0.0 | -67.9 | -63.1 | -131.0 | 1.0953 | 1.3793 | 2.4669 | 170.9 | 1.2478 | 127.6 | 1.8743 | 1.6736 | 134.8 | 172.0 |
| H-TON | Al1-O3 | 6.3 | -85.1 | -61.6 | -146.7 | 1.1713 | 1.2551 | 2.4193 | 171.2 | 1.2541 | 122.1 | 1.8299 | 1.6539 | 130.5 | 169.6 |
| H-TON | Al2-O3 | 0.0 | -80.4 | -61.1 | -141.5 | 1.1706 | 1.2489 | 2.4159 | 173.7 | 1.2533 | 120.9 | 1.8262 | 1.6459 | 130.9 | 176.0 |
| H-TON | Al3-O4 | 0.0 | -84.8 | -62.5 | -147.4 | 1.1739 | 1.2593 | 2.4299 | 174.0 | 1.2543 | 120.7 | 1.8267 | 1.6539 | 129.7 | 167.1 |
| H-TON | Al4-O6b | 3.5 | -49.9 | -65.0 | -114.9 | 1.0305 | 1.5636 | 2.5875 | 171.7 | 1.2407 | 152.2 | 1.8878 | 1.6804 | 132.5 | 172.5 |

**Table S5** Thermodynamically the most stable **acetonitrile** adsorption complexes for investigated aluminosilicate’s frameworks at different T-positions along with adsorption complexes formed at the most stable BAS if accessible.

| **Framework** | **T-site** | **E_rel_ (BAS)**  **[kJ.mol^-1^]** | **PBE**  **[kJ.mol^-1^]** | **D2**  **[kJ.mol^-1^]** | **PBE-D2**  **[kJ.mol^-1^]** | **O_B_-H_B_**  **[Å]** | **H_B_-N**  **[Å]** | **O_B_-N**  **[Å]** | **O_B_-H_B_-N**  **[deg]** | **N-C**  **[Å]** | **H_B_-N-C**  **[deg]** | **Al-O_B_**  **[Å]** | **Si-O_B_**  **[Å]** | **Al-O_B_-Si**  **[deg]** | **Al-O_B_-H_B_-Si**  **[deg]** |
| --- | --- | --- | --- | --- | --- | --- | --- | --- | --- | --- | --- | --- | --- | --- | --- |
| H-FAU | Al1-O1 | 0.0 | -77.3 | -19.0 | -96.3 | 1.0509 | 1.5349 | 2.5842 | 175.9 | 1.1613 | 165.8 | 1.8484 | 1.6661 | 125.0 | 175.7 |
| H-CHA | Al1-O2 | 6.0 | -79.9 | -25.4 | -105.3 | 1.0490 | 1.5513 | 2.5999 | 178.0 | 1.1616 | 174.8 | 1.8415 | 1.6685 | 132.5 | 173.6 |
| H-CHA | Al1-O4 | 0.0 | -71.4 | -30.8 | -102.2 | 1.0435 | 1.5707 | 2.6099 | 173.3 | 1.1616 | 172.9 | 1.8780 | 1.6777 | 130.1 | 160.8 |
| H-IFR | Al1-O8 | 10.7 | -77.1 | -26.4 | -103.5 | 1.0529 | 1.5249 | 2.5749 | 174.5 | 1.1610 | 170.2 | 1.8425 | 1.6654 | 125.6 | 170.3 |
| H-IFR | Al1-O5 | 0.0 | -47.5 | -30.5 | -78.0 | 1.0482 | 1.5666 | 2.6052 | 170.0 | 1.1617 | 161.8 | 1.8760 | 1.6703 | 125.7 | 141.7 |
| H-IFR | Al2-O4 | 5.3 | -81.5 | -25.7 | -107.2 | 1.0516 | 1.5401 | 2.5883 | 174.0 | 1.1617 | 165.6 | 1.8552 | 1.6697 | 132.3 | 177.3 |
| H-IFR | Al2-O1 | 0.0 | -66.9 | -26.9 | -93.8 | 1.0509 | 1.5421 | 2.5909 | 175.3 | 1.1617 | 162.3 | 1.8459 | 1.6715 | 129.3 | 165.8 |
| H-IFR | Al3-O1 | 0.8 | -63.4 | -26.8 | -90.2 | 1.0476 | 1.5564 | 2.6035 | 177.5 | 1.1616 | 176.2 | 1.8607 | 1.6675 | 128.8 | 167.6 |
| H-IFR | Al3-O5 | 0.0 | -53.2 | -33.7 | -86.9 | 1.0511 | 1.5556 | 2.6020 | 173.0 | 1.1614 | 175.0 | 1.8512 | 1.6800 | 126.9 | 144.1 |
| H-IFR | Al4-O8 | 3.3 | -76.3 | -25.3 | -101.7 | 1.0489 | 1.5404 | 2.5893 | 179.2 | 1.1612 | 169.6 | 1.8439 | 1.6638 | 124.3 | 168.8 |
| H-IFR | Al4-O9 | 0.0 | -63.5 | -27.9 | -91.3 | 1.0467 | 1.5621 | 2.6036 | 172.6 | 1.1614 | 172.0 | 1.8660 | 1.6801 | 130.2 | 160.4 |
| H-MOR | Al1-O4 | 0.0 | -76.8 | -25.0 | -101.9 | 1.0454 | 1.5618 | 2.6047 | 175.0 | 1.1615 | 176.1 | 1.8736 | 1.6795 | 132.0 | 171.6 |
| H-MOR | Al2-O7 | 0.0 | -76.5 | -24.1 | -100.5 | 1.0469 | 1.5509 | 2.5972 | 177.5 | 1.1612 | 178.9 | 1.8559 | 1.6725 | 130.6 | 178.0 |
| H-MOR | Al3-O3 | 0.0 | -49.2 | -57.4 | -106.5 | 1.0528 | 1.5361 | 2.5701 | 165.9 | 1.1642 | 145.2 | 1.8832 | 1.6818 | 127.4 | 145.0 |
| H-MOR | Al4-O7 | 0.0 | -74.4 | -27.2 | -101.5 | 1.0469 | 1.5580 | 2.6032 | 175.8 | 1.1614 | 173.5 | 1.8671 | 1.6698 | 127.0 | 157.1 |
| H-FER | Al1-O3 | 0.0 | -70.2 | -37.8 | -108.0 | 1.0486 | 1.5567 | 2.6039 | 176.2 | 1.1614 | 171.6 | 1.8672 | 1.6756 | 133.3 | 170.3 |
| H-FER | Al2-O2 | 0.0 | -66.1 | -39.4 | -105.4 | 1.0439 | 1.5661 | 2.5932 | 166.7 | 1.1619 | 163.2 | 1.8801 | 1.6756 | 130.6 | 171.3 |
| H-FER | Al3-O1 | 2.4 | -71.4 | -41.1 | -112.5 | 1.0445 | 1.5561 | 2.5890 | 168.9 | 1.1626 | 156.2 | 1.8687 | 1.6783 | 128.3 | 159.4 |
| H-FER | Al3-O7 | 0.0 | -60.6 | -41.6 | -102.3 | 1.0445 | 1.5539 | 2.5916 | 171.5 | 1.1612 | 171.1 | 1.8692 | 1.6834 | 133.1 | 174.7 |
| H-FER | Al4-O7 | 0.0 | -64.2 | -40.4 | -104.6 | 1.0476 | 1.5423 | 2.5834 | 171.7 | 1.1612 | 167.9 | 1.8887 | 1.6807 | 135.6 | 175.5 |
| H-TON | Al1-O3 | 6.3 | -78.9 | -40.0 | -118.8 | 1.0579 | 1.5182 | 2.5728 | 174.0 | 1.1617 | 158.8 | 1.8548 | 1.6725 | 130.0 | 173.9 |
| H-TON | Al2-O3 | 0.0 | -75.9 | -40.2 | -116.2 | 1.0560 | 1.5153 | 2.5681 | 174.2 | 1.1615 | 158.0 | 1.8518 | 1.6677 | 129.3 | 173.9 |
| H-TON | Al3-O4 | 0.0 | -77.7 | -39.9 | -117.6 | 1.0515 | 1.5420 | 2.5889 | 173.1 | 1.1615 | 170.4 | 1.8494 | 1.6727 | 132.0 | 171.5 |
| H-TON | Al4-O6b | 3.5 | -57.5 | -45.4 | -102.9 | 1.0363 | 1.6232 | 2.6332 | 163.5 | 1.1619 | 173.5 | 1.8816 | 1.6819 | 133.4 | 172.9 |

| **a** | **b** |
| --- | --- |
| **** | **** |
| **c** | **d** |
| **** | **** |
| **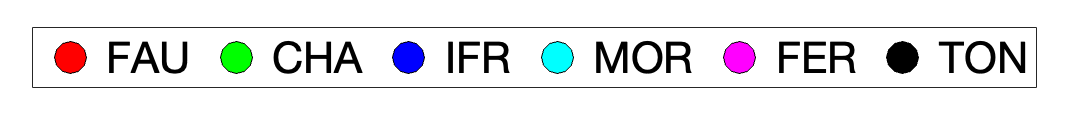** | |

**Figure S5** Correlation of *f*_dep_ descriptor and with O_B_-H_B_ bond lengths upon adsorption of (a) ammonia, (b) formamide, (c) acetone and (d) acetonitrile.

**Figure S6** Correlation between deprotonation metric f_dep_ as defined in Eq.2 and PBE adsorption energies for the most stable adsorption complexes.

| **a** | **b** |
| --- | --- |
| **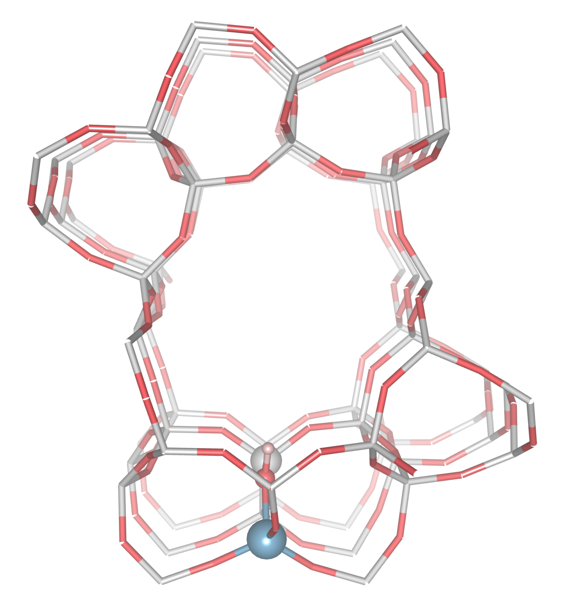** | **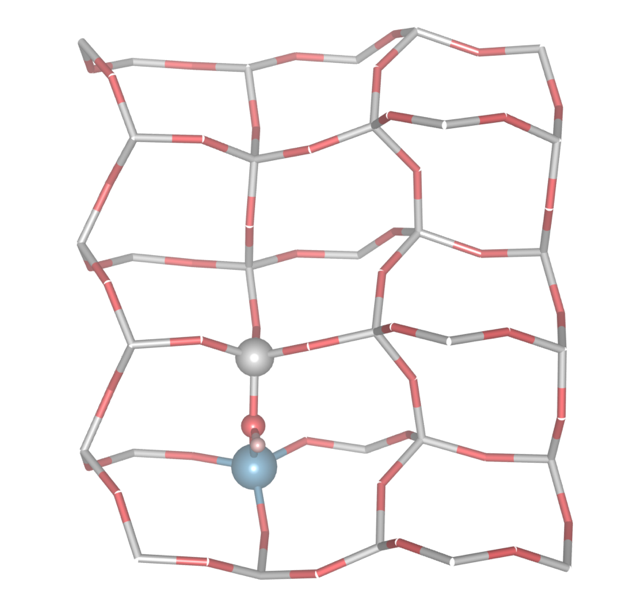** |

**Figure S7** **TON**/Al4 BAS (a) view along the main channel, (b) view from the top.^1^

**References**

1 Momma, K. & Izumi, F. VESTA 3 for three-dimensional visualization of crystal, volumetric and morphology data. *J. Appl. Crystallogr.* **44**, 1272-1276, doi:10.1107/s0021889811038970 (2011).

2 Jones, A. J. & Iglesia, E. The Strength of Brønsted Acid Sites in Microporous Aluminosilicates. *ACS Catalysis* **5**, 5741-5755, doi:10.1021/acscatal.5b01133 (2015).

3 Trachta, M., Bulanek, R., Bludsky, O. & Rubes, M. Bronsted acidity in zeolites measured by deprotonation energy. *Sci Rep* **12**, 7301, doi:10.1038/s41598-022-11354-x (2022).

4 Rybicki, M. & Sauer, J. Acid strength of zeolitic Brønsted sites—Dependence on dielectric properties. *Catal. Today* **323**, 86-93, doi:10.1016/j.cattod.2018.04.031 (2019).

5 Rubeš, M., Trachta, M., Vaculík, J., Bulánek, R. & Bludský, O. The analysis of the BAS OH band in zeolites. *Microporous Mesoporous Mater.*, 112052 (2022).

6 Schroeder, C., Zones, S. I., Hansen, M. R. & Koller, H. Hydrogen Bonds Dominate Bronsted Acid Sites in Zeolite SSZ-42: A Classification of Their Diversity. *Angew. Chem. Int. Ed. Engl.* **61**, e202109313, doi:10.1002/anie.202109313 (2022).

7 Grajciar, L., Arean, C. O., Pulido, A. & Nachtigall, P. Periodic DFT investigation of the effect of aluminium content on the properties of the acid zeolite H-FER. *Phys. Chem. Chem. Phys.* **12**, 1497-1506, doi:10.1039/b917969k (2010).

8 van Santen, R. A. & Kramer, G. J. Reactivity Theory of Zeolitic Broensted Acidic Sites. *Chem. Rev.* **95**, 637-660, doi:10.1021/cr00035a008 (1995).
